# Attenuating vascular stenosis-induced astrogliosis preserves white matter integrity and cognitive function

**DOI:** 10.1186/s12974-021-02234-8

**Published:** 2021-08-28

**Authors:** Qian Liu, Mohammad Iqbal H. Bhuiyan, Ruijia Liu, Shanshan Song, Gulnaz Begum, Cullen B. Young, Lesley M. Foley, Fenghua Chen, T. Kevin Hitchens, Guodong Cao, Ansuman Chattopadhyay, Li He, Dandan Sun

**Affiliations:** 1 Department of Neurology, West China Hospital, Sichuan University, Chengdu, 610041 Sichuan China; 2 Department of Neurology, University of Pittsburgh, Pittsburgh, Pennsylvania 15213 USA; 3 Pittsburgh Institute for Neurodegenerative Disorders, University of Pittsburgh, Pittsburgh, Pennsylvania 15213 USA; 4 Animal Imaging Center, University of Pittsburgh, Pittsburgh, Pennsylvania 15213 USA; 5 Department of Neurobiology, University of Pittsburgh, Pittsburgh, Pennsylvania 15213 USA; 6 VA Pittsburgh Healthcare System, Geriatric Research Education and Clinical Center, Pittsburgh, Pennsylvania 15240 USA; 7 Molecular Biology-Information Service, Health Sciences Library System, University of Pittsburgh, Pittsburgh, Pennsylvania 15261 USA

**Keywords:** Hypoperfusion, Demyelination, Gliosis, Na^+^/H^+^ exchanger, Vascular dementia

## Abstract

**Background:**

Chronic cerebral hypoperfusion (CCH) causes white matter damage and cognitive impairment, in which astrogliosis is the major pathology. However, underlying cellular mechanisms are not well defined. Activation of Na^+^/H^+^ exchanger-1 (NHE1) in reactive astrocytes causes astrocytic hypertrophy and swelling. In this study, we examined the role of NHE1 protein in astrogliosis, white matter demyelination, and cognitive function in a murine CCH model with bilateral carotid artery stenosis (BCAS).

**Methods:**

Sham, BCAS, or BCAS mice receiving vehicle or a selective NHE1 inhibitor HOE642 were monitored for changes of the regional cerebral blood flow and behavioral performance for 28 days. Ex vivo MRI-DTI was subsequently conducted to detect brain injury and demyelination. Astrogliosis and demyelination were further examined by immunofluorescence staining. Astrocytic transcriptional profiles were analyzed with bulk RNA-sequencing and RT-qPCR.

**Results:**

Chronic cerebral blood flow reduction and spatial working memory deficits were detected in the BCAS mice, along with significantly reduced mean fractional anisotropy (FA) values in the corpus callosum, external capsule, and hippocampus in MRI DTI analysis. Compared with the sham control mice, the BCAS mice displayed demyelination and axonal damage and increased GFAP^+^ astrocytes and Iba1^+^ microglia. Pharmacological inhibition of NHE1 protein with its inhibitor HOE642 prevented the BCAS-induced gliosis, damage of white matter tracts and hippocampus, and significantly improved cognitive performance. Transcriptome and immunostaining analysis further revealed that NHE1 inhibition specifically attenuated pro-inflammatory pathways and NADPH oxidase activation.

**Conclusion:**

Our study demonstrates that NHE1 protein is involved in astrogliosis with pro-inflammatory transformation induced by CCH, and its blockade has potentials for reducing astrogliosis, demyelination, and cognitive impairment.

**Supplementary Information:**

The online version contains supplementary material available at 10.1186/s12974-021-02234-8.

## Background

Vascular contributions to cognitive impairment and dementia (VCID) have been identified as an important vascular pathologic process in the initiation and progression of vascular dementia and Alzheimer’s disease (AD), which together account for approximately 60–80% of dementia worldwide [1, 2]. Chronic cerebral hypoperfusion (CCH) resulting from either large or small cerebral vessel diseases (such as carotid atherosclerosis or arteriosclerosis) causes subsequent white matter lesions (WMLs) and cognitive impairment and dementia [3–5]. Carotid stenosis-induced cerebral hypoperfusion is an independent risk factor for WMLs and cognitive impairment [5–7], with severe stenosis causing pronounced cognitive impairment [6, 7]. Characteristic pathology of VCID includes white matter lesion, cerebral atrophy, gliosis, and endothelial damage, in part resulting from oxidative stress and neuroinflammation [3, 8]. However, the underlying cellular mechanisms for hypoperfusion-induced WMLs and VCID are not well understood, and there is an urgent need to better understand its pathogenesis and develop therapies for the prevention and/or treatment of VCID.

Reactive glial cells, chronic inflammation, and oxidative stress are closely correlated with neurodegeneration and cognitive impairment [9, 10]. Glial activation was detected in the early stage of AD patients, and the subsequent glia-mediated inflammatory process was suggested to lead to cognitive impairment progression [11, 12]. Serum and cerebrospinal fluid inflammatory biomarkers in older adults were significantly associated with cerebral small vessel disease and cognitive decline [13, 14]. Experimental data from a chronic hypoperfusion-induced murine VCID model showed that gliosis and a sustained inflammatory response play an important role in white matter lesion development [15]. Activation of astrocytic Na^+^/H^+^ exchanger 1 (NHE1) causes hypertrophy and swelling of reactive astrocytes after acute brain injury [16, 17]. In reactive astrocytes or activated microglia, NHE1 protein plays an important role in regulating intracellular pH (pH_i_) homeostasis by extrusion of H^+^ in exchange for Na^+^ [18–21]. NHE1-mediated H^+^ extrusion promotes sustained NADPH oxidase (NOX) function and pro-inflammatory responses by maintaining an alkaline pH_i_ in microglia [19]. Moreover, increases in intracellular Na^+^ in reactive astrocytes following NHE1 protein activation trigger a reversal of Na^+^/Ca^2+^ exchange and stimulation of the Ca^2+^-dependent signal pathways, including the release of glutamate and cytokines from astrocytes [20, 22]. In a mouse neonatal hypoxia–ischemia brain injury model, pharmacological inhibition of NHE1 by its potent inhibitor HOE642 reduced corpus callosum white matter damage and improved cognitive function [23]. These studies demonstrated that activation of glial NHE1 protein is involved in gliosis and neuroinflammation after acute ischemic or hypoxia neonatal brain injury. However, whether NHE1 protein activation plays a role in astrogliosis in chronic hypoperfusion-induced brain injury remains unknown. In this study, using a well-established murine bilateral carotid artery stenosis (BCAS) model for CCH, we detected increased GFAP^+^ astrocytes and Iba1^+^ microglia exhibiting NHE1 protein expression. Post-BCAS administration of the selective NHE1 inhibitor HOE642 significantly decreased astrogliosis, preserved white matter and hippocampus integrity, and improved cognitive function by preventing astrocytic ROS production and inflammatory transcriptomes. These findings revealed the potential of pharmacological blockade of NHE1 protein in reducing cerebral hypoperfusion-induced chronic brain injury and cognitive impairment.

## Methods

### Materials

Vendor and material information were included in the **Supplemental data****.**

### Animals and BCAS model

All animal studies were approved by the University of Pittsburgh Medical Center Institutional Animal Care and Use Committee, which adhere to the National Institutes of Health Guide for the Care and Use of Laboratory Animals, and reported in accordance with the Animal Research: Reporting In Vivo Experiments (ARRIVE) guidelines [24]*.* All efforts were made to minimize animal suffering and the number of animals used.

C57BL/6J male mice (aged 9–12 weeks, weighing 25 to 30 g) were subjected to sham or BCAS surgery procedures. To induce BCAS, mice were anesthetized with 1.5% isoflurane in 70% N_2_O and 30% O_2_ and placed in the supine position. Body temperature was maintained at 36.5 ± 0.5 °C with a heating pad. Through a midline incision, the common carotid artery was carefully exposed and isolated from the vagus nerve and surrounding tissues. After gently lifting the carotid artery, the steel spring microcoil (0.18-mm internal diameter, WUXI SAMINI SPRING Co., Ltd.) was twined by rotating around the common carotid artery and placed below the carotid bifurcation. After suturing, 50 μl of 0.25% bupivacaine hydrochloride was placed on the top of the incision for local infiltration anesthesia. Animals were returned to the normal cage to recover with free access to food and water.

### Administration of NHE1 inhibitor HOE642

BCAS mice were randomly allocated to receive either DMSO+saline vehicle (0.5% DMSO in saline, *n* = 8) or HOE642 (0.3mg/kg/day, *n* = 8) via intraperitoneal (i.p.) injection daily from 3–30 days after BCAS surgery. A separate group of mice (*n* = 9) was implanted with an osmotic mini-pump (Alzet, type 1004, Durect corporation, Cupertino, CA) to constantly deliver HOE642 (0.3 mg/kg/day at a rate of 208.3 ng/kg/min) from onset to 28 days after BCAS surgery. Naïve control (*n* = 3) or sham control mice (which underwent bilateral common carotid artery isolation procedures without micro-coils placement, *n* = 3–5) and 3 BCAS mice received no treatments.

### Cerebral blood flow measurement

CBF in mice was measured using a two-dimensional laser speckle contrast analysis system (PeriCam PSI High Resolution with PIMSoft, Perimed, Sweden) as described before [16]. After mice were anesthetized with 1.5% isoflurane in 70% N_2_O and 30% O_2_, a midline incision was made in the scalp and the exposed skull surface was cleaned with sterile normal saline. Raw speckle images of regions of interest (ROIs) covering the parietal lobe in each hemisphere were taken with a camera placed 10 cm above the skull. Regional CBF (rCBF) values (arbitrary perfusion units) were measured for same size areas in both parietal lobes at baseline, 5–10 min, 7 days, and 30 days post-surgery. The percentage change of rCBF at each post-surgery time point was calculated by comparing the mean signal intensity to that of the baseline. Since isoflurane might have effects on rCBF [25, 26], we have maintained isoflurane administration time and the concentration consistent for each animal to avoid potential confounding effects in each group. Additionally, body temperature was maintained at 36.5 ± 0.5 °C with a heating pad during surgery.

### Neurological behavioral function tests

Neurologic function tests in mice were conducted in a blinded manner at 28–30 days after surgery, which includes the open field (OF) test to assess the locomotor activities and the Y-maze test to assess spatial working memory. In the Y maze test*:* each mouse was placed in a clear polycarbonate arena in the shape of a Y consisting of 3 arms that were 33.65 cm long, 15 cm high, and 6 cm wide (Muromachi Kikai, Tokyo, Japan), and the mouse’s behavior during free exploration of the Y maze was recorded for 8 min [27]. Behavioral tracking software (Noldus Ethovision XT) was used to analyze spontaneous alterations. The percentage of spontaneous alterations was calculated as the ratio of actual to possible alterations [defined as the frequency of spontaneous alteration behavior/(the total number of arm entries − 2) × 100]. In the OF test: each mouse was placed in the center of a square arena of open field apparatus (40 × 40 × 40 cm; Omnitech Electronics) within environmental control chamber (60 × 64 × 60 cm; Omnitech Electronics). Total distance traveled (in cm), vertical activity (rearing measured by counting the number of photobeam interruptions), and margin time (time spent in the periphery of the arena) were recorded using behavioral tracking software (Fusion, Omnitech Electronics). Data were collected for 60 min.

### Magnetic resonance imaging DTI of ex vivo brain

Post neurological behavioral tests, mice were anesthetized with 3% isoflurane in 70% N_2_O and 30% O_2_, and transcardially perfused with 4% paraformaldehyde (PFA) and decapitated [28]. Brains were maintained within the skull to avoid anatomical deformation and fixed in 4% PFA overnight, then stored in PBS solution at 4 °C. MRI was performed at 500 MHz using a Bruker AV3HD 11.7 T/89 mm vertical bore small animal MRI scanner, equipped with a 20-mm quadrature radiofrequency (RF) coil and Paravision 6.0.1 software (Bruker Biospin, Billerica, MA). Following positioning and pilot scans, T2-weighted images (T2WI) were acquired using a Rapid Acquisition with Relaxation Enhancement (RARE) sequence, with the following parameters: Echo Time/Repetition Time (TE/TR) = 20/4000 ms, averages = 8, 160 × 160 matrix, 25 slices with a 0.5 mm slice thickness, a RARE factor = 4, and a field of view (FOV) of 16 × 16 mm. Hippocampal atrophy or signal abnormality (low or high signal intensity) were identified as injury on T2-weighted images by one expert in small animal MRI imaging. A diffusion tensor imaging (DTI) data set covering the entire brain was collected using a multislice spin echo sequence with five reference and 30 non-collinear diffusion-weighted images with the following parameters: TE/TR = 22/2800 ms, two averages, matrix size = 160 × 160, field of view = 16 × 16 mm, 25 axial slices, slice thickness = 0.5 mm, *b* value = 3000 s/mm^2^, and *Δ*/*δ* = 11/5 ms. MRI DTI data were analyzed with DSI Studio (http://dsistudio.labsolver.org/). In a blinded manner, regions of interest (ROIs) were drawn from the corpus callosum (CC), external capsule (EC), and hippocampus. Fractional anisotropy (FA), axonal diffusivity (AD), radial diffusivity (RD), and mean diffusivity (MD) values were determined for each ROI from identical consecutive sections of DTI images.

### Immunofluorescence staining image collection and IMARIS 3D morphological analysis of astrocytes

Post-MRI PFA-fixed brains were equilibrated in 30% sucrose at 4 °C and coronal brain sections (25 μm) were prepared using a cryostat (Leica SM2010R, Biosystems). After incubation with a blocking buffer for 1 h at room temperature, sections were incubated with primary antibodies against MBP (Rabbit, 1:800 dilution), NF-200 (Rabbit, 1:800 dilution), NHE1 (Rabbit; 1:100 dilution), GFAP (Mouse; 1:200 dilution), Iba1 (Goat; 1:600 dilution), NeuN (Mouse; 1:200 dilution), Phospho-p47phox (Rabbit; 1:100 dilution), or Lcn2 (Rat; 1:200 dilution) in blocking solution at 4 °C overnight. These brain sections were washed and incubated with secondary antibodies (1:200 dilution) for 1 h at room temperature. Subsequently, these sections were washed and incubated with DAPI (4,6-diamino-2-phenylindole, 1:1000 in 0.1M PBS) for 15 min at room temperature. Sections were then mounted on slides with a mounting medium. Fluorescence images of hippocampus overview were obtained under 10× objective by the Olympus IX83 inverted microscope (Olympus, Tokyo, Japan) and processed with Olympus cellSens Dimension software (version 2.3, Olympus). The fluorescence images from a 40× oil-immersion objective were captured using the Nikon A1R confocal microscope (Nikon, Tokyo, Japan) with NIS-Elements AR software (version 4.51, Nikon). Images were obtained from identical slides positions using identical digital imaging acquisition parameters. Numbers of positively stained cells and intensity of immunoreactivity were quantified from the 40× oil-immersion objective images using the ImageJ software. Intensity of immunoreactivity was quantified by measuring the mean gray values and the results were expressed in arbitrary units.

For the 3D reconstruction and morphological quantitative analysis of reactive astrocytes, Bitplane Imaris software (Version 9.7.2, Bitplane, Zurich, Switzerland) was used. *Z*-stack images of GFAP^+^ astrocytes (18-μm depth, 1.69-μm steps, × 40 magnification) were taken using a Nikon A1R confocal microscope (1024 × 1024 pixel, pixel size 0.16 μm). Raw images were converted using IMARIS converter (Version 9.7.2, Oxford Instruments). Images were subjected to surface and filament reconstruction based on GFAP immunostaining in three dimensions (3D). Surface reconstruction parameters were set to appropriately label all GFAP^+^ astrocytes. The astrocyte processes and the voxels within one stack were rendered into 3D objects and the volume was analyzed. The cell body volume of the obtained objects was expressed as summated soma volume. The IMARIS Filament module was used to quantify morphological changes of astrocytic processes using the following endpoints: summarized process volume, mean diameter, and total terminal points of process. All images used for analysis were taken with the same confocal settings.

### Astrocyte isolation and RNA extraction for RNA sequencing

In order to investigate the transcriptomic changes of astrocytes in response to BCAS and post-BCAS HOE642 treatment, sham, BCAS+Veh, and BCAS+HOE642 (i.p.) mice were harvested for RNA-seq (4 mice/group) and RT-qPCR (6 mice/group). At 30 days post-surgery, brains were removed and rapidly dissected in an ice-cold D-PBS solution. Single-cell suspensions were prepared from both hemispheres (without the cerebellum and brain stem) using the Adult Brain Dissociation Kit (Miltenyi Biotec, Germany), as described previously [29]. Hemispheres were separated in an enzyme mixture solution with gentle MACS Octo Dissociator at 37 °C for 30 min. Digested tissues were filtered through a 70-μm MACS Smart Strainer and followed by several steps of centrifugation to obtain a single-cell suspension. Astrocytes were further isolated by magnetic bead separation using anti-ACSA-2 microbead kit (Miltenyi Biotec, Germany). The RNA of ACSA2^+^ astrocytes was extracted using the RNeasy Micro kit (Qiagen, 74004) following the manufacturer’s protocol. The resulting RNA was eluted with RNase-free water and stored at – 80 °C. Samples were sequenced on an Illumina NovaSeq 6000 (PE150) using Illumina TruSeq stranded mRNA kit for library preparation. Total RNA (~ 300 ng) was used as input for library preparation.

### Bioinformatic data analysis

RNA-Seq data were analyzed following the instruction of CLC genomics Workbench 21 (CLC bio, Aarhus, Denmark) [29]. Briefly, quality control was conducted for the paired-end reads in FASTQ format, before mapping against the mouse reference genome GRCm38 (mm10) using default parameters of the “RNA-Seq Analysis” tool. Gene and transcript annotations were completed with Ensembl (release V103). Differentially expressed genes (DEGs) were identified between sham, BCAS+Veh, and BCAS+HOE (i.p.) groups using the “Differential Expressions for RNA seq” tool. Genes with a *p* value ≤ 0.05 and fold change (FC) ≥ 1.5 or ≤ − 1.5 were identified as differentially expressed genes. QIAGEN’s Ingenuity Pathway Analysis (IPA®, QIAGEN Redwood City, www.qiagen.com/ingenuity) was used to identify statistically enriched biological pathways associated with the differentially expressed genes. Statistical significance was calculated using the right-tailed Fisher’s exact probability tests; biological pathways showing *p* value < 0.05 were considered statistically significant. The activity status of pathways was determined by calculating the activity *Z*-score, a statistical measure of how closely the gene expression pattern present in the query dataset compares to the expected pattern based on the literature findings [30]. A positive score indicates an overall increase in the pathway activity, whereas a negative value indicates an overall decrease in activity. The IPA Comparison Analysis tool was used to compare pathway enrichment analysis results generated from the multiple datasets used in our study. A *p* value < 0.05 and a *Z*-score ≥ 2 were set as the thresholds for statistical significance. Gene ontology (GO) analyses were conducted for biological processes, using the Database for Annotation Visualization and Integrated Discovery (DAVID; https://david.ncifcrf.gov/) [31], with *p* value < 0.05 and a gene count ≥ 2 as the thresholds to indicate a statistically significant difference.

### RT-qPCR analysis

RNA was extracted from MACS-isolated astrocytes using the RNeasy Micro kit (Qiagen, 74004) following the manufacturer’s instructions. RNA was quantified by measuring absorbance with spectrophotometer ND-1000 (NanoDrop). Reverse transcription was performed using the iScript Reverse Transcription Supermix (Bio-Rad) according to the manufacturer’s protocol. All RNA isolated from cell pellets was converted into cDNA. Quantitative RT-PCR was performed using iTaq Universal SYBR Green Supermix (Bio-Rad) on a CFX 96 Touch Real-Time PCR Detection System. All relative gene expression analyses were performed using the 2^−ΔΔCt^ method with duplicate reactions for each evaluated gene. Following primer sequences were used: *Hprt* (housekeeping gene), forward: GCC TAA GAT GAG CGC AAG TTG, reverse: TAC TAG GCA GAT GGC CAC AGG; *Ptgs2*, forward: TGA GCA ACT ATT CCA AAC CAGC, reverse: GCA CGT AGT CTT CGA TCA CTA TC; *Nos3*, forward: TCA GCC ATC ACA GTG TTC CC, reverse: ATA GCC CGC ATA GCG TAT CAG; *Lnc2*, forward: TGG CCC TGA GTG TCA TGTG, reverse: CTC TTG TAG CTC ATA GAT GGT GC; *Mmp9* forward: GGA CCC GAA GCG GAC ATT G, reverse: CGT CGT CGA AAT GGG CAT CT. The data were normalized to *Hprt* as a reference gene.

### Statistical analysis

Mice were coded with randomized numbers and outcome assessments were performed by investigators who were blinded to the treatment conditions. A total of 69 mice were used and all data were included except two outlier samples excluded in RNA-seq bioinformatics analysis after inspection of a PCA bi-plot and data. Normality was assessed by the Shapiro–Wilk test. Data were presented as mean and standard deviation (SD) if data were normally distributed, or reported as median and quartiles if data were not normally distributed. Statistical significance was determined by Student’s *t*-test or one-way analysis of variance (ANOVA) followed by Bonferroni post hoc test. The repeated measured values within groups were analyzed by repeated measure ANOVA followed by Bonferroni’s post hoc test. The GraphPad Prism software was used for statistical analyses (GraphPad Software, Inc., CA, USA). The Pearson correlation analysis and ANOVA analysis followed by the LSD post hoc test for DTI metrics were performed using SPSS 24 (SPSS Inc., Chicago, Ill., USA). A *p* value < 0.05 was considered statistically significant.

## Results

### Effects of NHE1 blockade on BCAS-induced changes in rCBF and cognitive function impairment

**Fig. 1:**
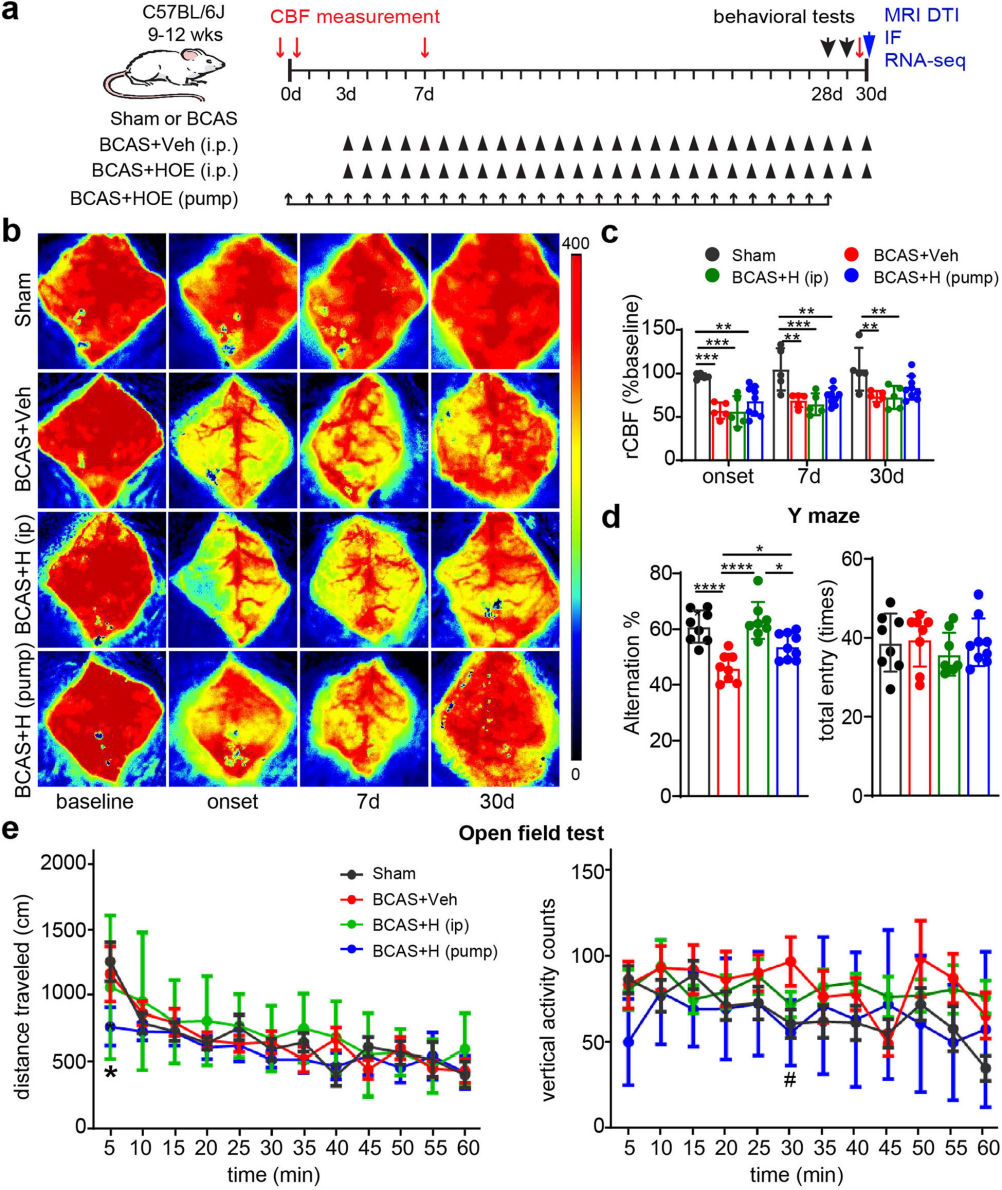
Pharmacological inhibition of NHE1 protein in BCAS mice improved their spatial working memory. **a** Experimental protocol. C57BL/6J mice underwent sham or BCAS surgery. BCAS mice received either saline vehicle (Veh) or HOE642 (0.3mg/kg/day) via intraperitoneal injection (i.p.) for 3–30 days or via mini-pump for 0-28 days. **b** Representative images of regional cerebral blood flow (rCBF). **c** PeriCam PSI analysis of rCBF. Data are presented as mean ± SD. *n* = 5–9/group. *p < 0.05, **p < 0.01, ***p < 0.001. **d** The percentage of spontaneous alternation and total entry in Y maze test. *n* = 8–9/group. *p < 0.05, ****p < 0.0001. **e** Open field test. Data are represented as mean ± SD. *n* = 8–9/group. *p < 0.05 vs. sham. ^#^*p* < 0.05 vs. Veh

C57BL/6J mice were subjected to sham or BCAS surgery, or with subsequent treatment regimens including BCAS+Veh (i.p.), BCAS+HOE642 (i.p.), or BCAS+HOE642 (pump) (Fig. 1a). PeriCam laser speckle contrast analysis shows that sham mice displayed no significant changes in rCBF from prior to surgery through 30 days post-surgery (*p* > 0.05, Fig. 1b, c). In contrast, the BCAS+Veh mice and BCAS+HOE (i.p.) mice displayed ~ 45% rCBF reduction at the onset of BCAS, which gradually recovered to ~ 75% of baseline by 30 days post-surgery **(***p* < 0.05, Fig. 1b, c). However, the BCAS+HOE (pump) mice showed a trend of less rCBF reduction at onset, 7 days, or 30 days after BCAS surgery, compared to the BCAS+HOE (i.p.) group, but did not reach statistical significance (*p* > 0.05, Fig. 1b, c). No differences in rCBF were detected between the BCAS+Veh mice and BCAS+HOE (i.p.) mice (*p* > 0.05, Fig. 1b, c).

We then evaluated neurological function changes in these mice with Y maze and OF tests. In the Y maze test, no differences in total entry counts were detected among all four testing groups, indicating similar locomotor activity in these mice (*p* > 0.05, Fig. 1d). However, a significantly lower alternation rate, indicative of spatial working memory deficit [32], was observed in the BCAS+Veh group, compared with the sham group (*p* < 0.05, Fig. 1d). Interestingly, the BCAS+HOE (i.p.) group displayed similar spontaneous alternation rates as the sham group, showing no spatial working memory deficit. The BCAS+HOE (pump) mice performed significantly better than the BCAS+Veh group but worse than the BCAS+HOE (i.p.) group (*p* < 0.05, Fig. 1d). In the OF test, no significant differences in distance traveled or margin time (data not shown) were detected among the four testing groups (Fig. 1e). However, the BCAS+Veh group exhibited a trend of higher vertical activities, compared with the sham group, but did not reach statistical significance (*p* > 0.05). The BCAS+HOE (pump) mice showed occasional lower locomotor activity, e.g., less traveled distance than sham mice at the initial 5 min and less vertical activity at 30 min than the BCAS+Veh mice (*p* < 0.05, Fig. 1e). Taken together, these data demonstrated that BCAS-induced cerebral hypoperfusion in mice impaired their spatial working memory. Pharmacological blockade of NHE1 protein with NHE1 inhibitor HOE642 (daily i.p. injection or continuous mini pump delivery) effectively prevented or attenuated BCAS-induced cognitive function impairment.

### Pharmacological inhibition of NHE1 protein attenuated BCAS-induced white matter tract damage

**Fig. 2:**
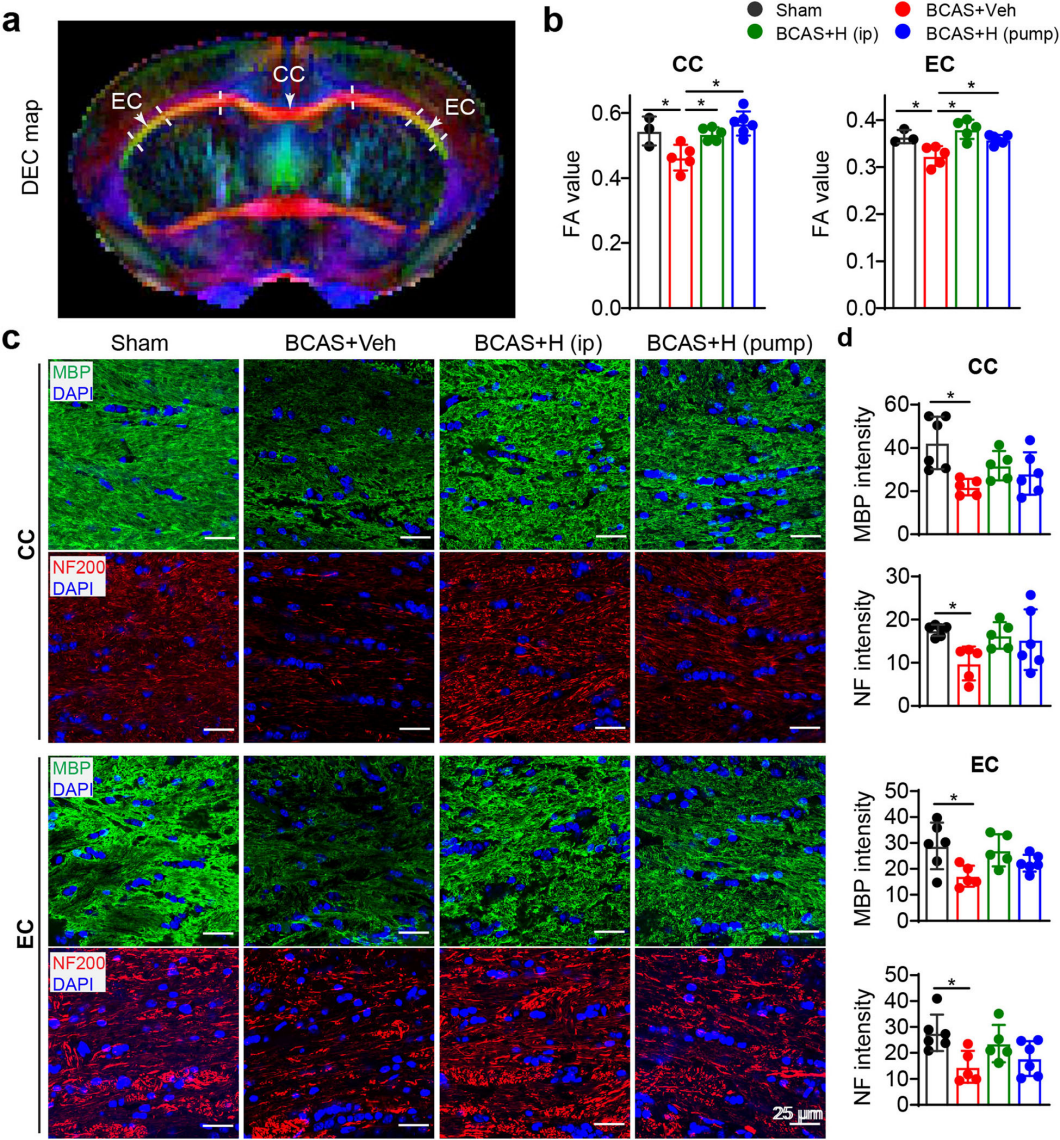
Blocking NHE1 with inhibitor HOE642 in BCAS mice ameliorated white matter damage. **a** Representative DEC maps showing corpus callosum (CC) and external capsule (EC) for DTI analysis. At 30 days post-BCAS following neurological function analysis in Fig. 1d, mice were sacrifized for ex vivo brain MRI DTI and subseqent immunoflurorescence staining. **b** Quantitative analysis of mean FA values in CC and EC. Data are presented as mean ± SD. sham group: *n* = 3, BCAS+Veh and BCAS+HOE (i.p. or pump): *n* = 5–6. *p < 0.05. **c** Representative images of MBP or NF staining in CC and EC. However, due to lack of optimal anti-MBP and NF200 antibodies for double labeling, the images in Fig. 2C were obtained from anti-MBP or anti-NF200 staining, respectively. **d** Quantitative analysis of immunofluorescent intensity of MBP and NF in CC and EC, respectively. Data are presented as mean ± SD. *n* = 5–6/group. *p < 0.05

To understand the mechanisms underlying neurological function protection in the HOE642-treated mice, ex vivo MRI studies were performed on brains from these four groups of mice (sham, BCAS+Veh, and BCAS+HOE mice (i.p., or pump). Representative directionally encoded color (DEC) maps, FA maps, and T2-weighted images showed no obvious white matter lesions in the four groups (**supplemental Fig S****1****a**). However, DTI metrics measurement and analysis of white matter tracts (Fig. 2a), such as corpus callosum (CC) and external capsule (EC), revealed an average of 15% reduction in the mean FA values in CC (*p* < 0.05, Fig. 2b) and 11.2% reduction in EC (*p* < 0.05, Fig. 2b) in the BCAS+Veh brains, compared to sham brains. In contrast, the BCAS+HOE-treated brains (i.p. or pump) showed significantly higher FA values in CC and EC than the BCAS+Veh brains (*p* < 0.05, Fig. 2b). These non-biased DTI findings indicate that pharmacological blockade of NHE1 protein preserved white matter integrity, which may contribute to improved neurological function outcomes. This speculation is supported by immunofluorescence staining of these brains for assessment of demyelination, shown by loss of myelin basic protein (MBP) and axonal neurofilament (NF-200) expressions. Due to a lack of optimal anti-MBP and NF200 antibodies for double labeling, the images in Fig. 2c were obtained from anti-MBP or anti-NF200 staining with DAPI respectively. Compared to sham or naïve brains (**supplemental Fig. S****2**), reduction of MBP (40–48%) and NF expression (44–47%) was detected in CC and EC of the BCAS+Veh brains (*p* < 0.05, Fig. 2c, d) or the BCAS mice (**supplemental Fig. S****2**)**.** In contrast, the BCAS+HOE-treated mice (i.p. or pump) showed a trend of higher MBP or NF-200 expression in CC and EC than the BCAS+Veh mice (*p* > 0.05**,** Fig. 2c, d). The FA values were highly correlated with NF expression in CC and EC (with the Pearson coefficient *r* = 0.66–0.80, *p* < 0.05, **Table S****1**). Taken together, these results demonstrate that inhibition of NHE1 activity with HOE642 ameliorated BCAS-induced demyelination and axonal damage in white matter tracts.

### Pharmacological inhibition of NHE1 protein reduced BCAS-induced astrogliosis in white matter tissues

**Fig. 3:**
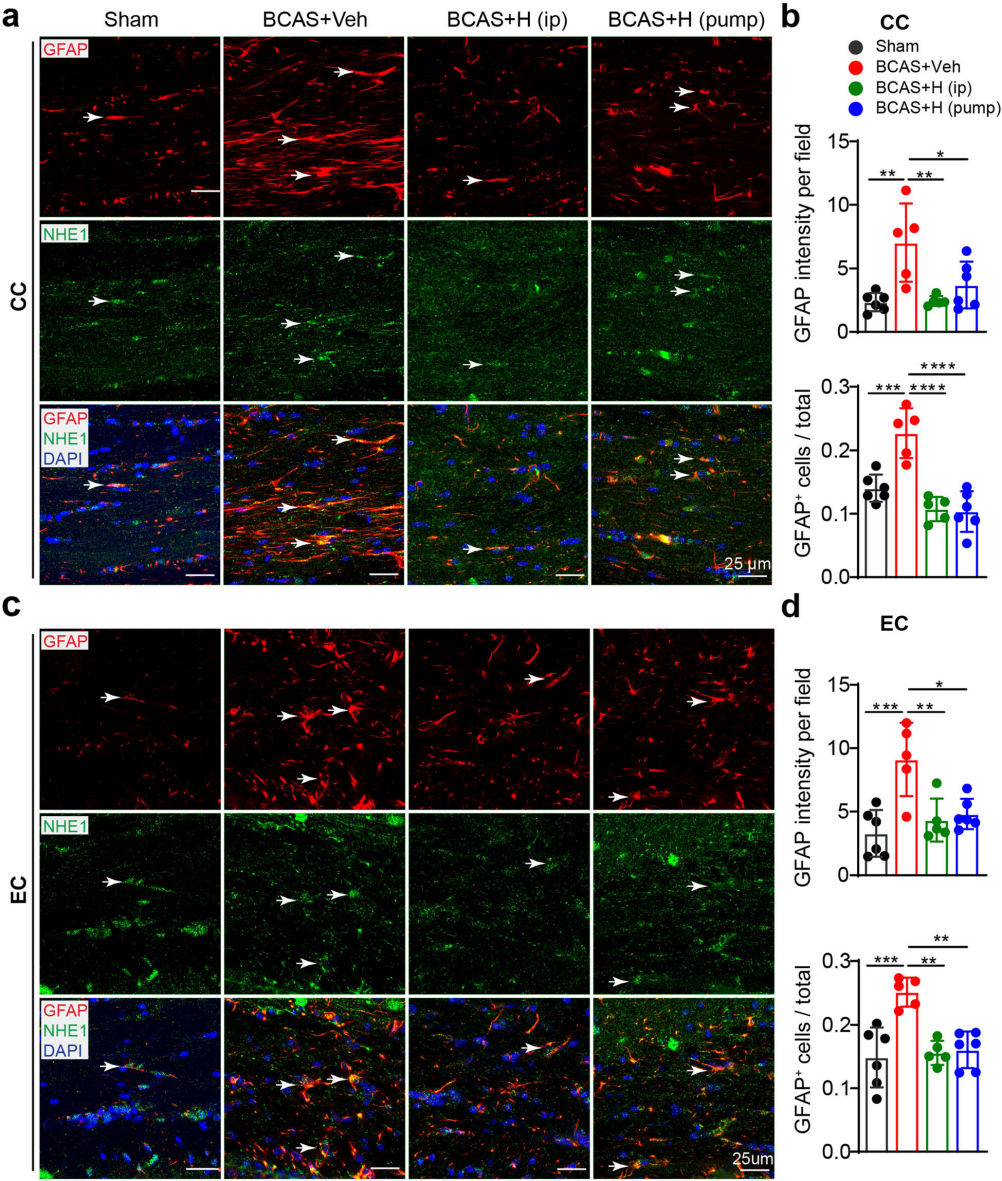
Pharmacological inhibition of NHE1 suppressed BCAS-induced formation of reactive astrogliosis in white matter tracts. **a**, **c** Representative images of immunostaining of GFAP and NHE1 protein in CC (**a**) and EC (**c**) of mice at 30 days post-surgery. Arrow: GFAP or NHE1 protein expression in GFAP^+^ cells. **b**, **d** Quantitative analysis of GFAP^+^ cell counts as well as GFAP intensity in CC (**b**) or EC (**d**). Data are presented as mean ± SD. *n* = 5–6/group. *p < 0.05, **p < 0.01, ***p < 0.001, ****p < 0.0001

**Fig. 4:**
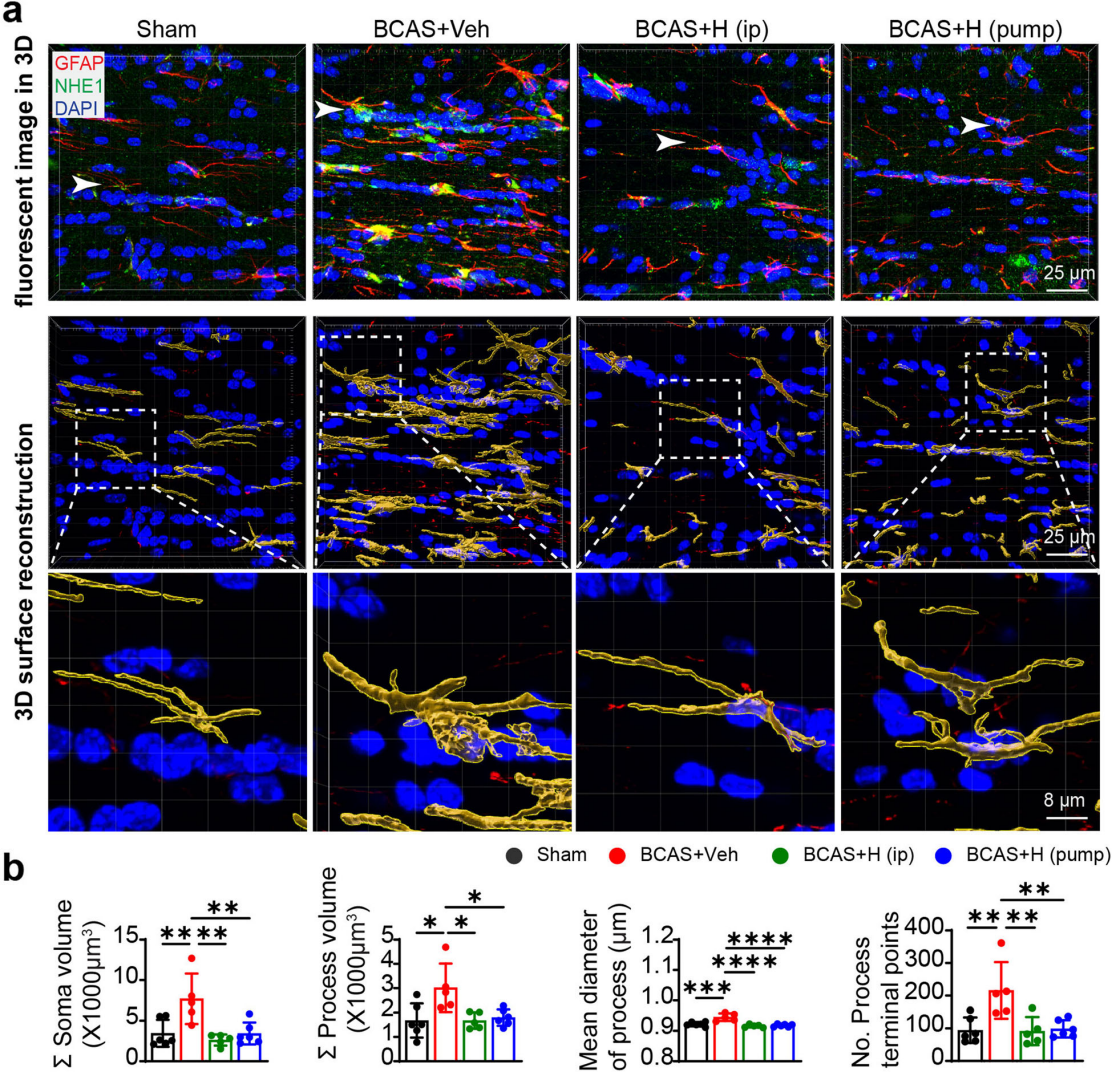
Inhibition of NHE1 by HOE642 suppressed BCAS-induced hypertrophy of reactive astrocytes in white matter tracts. **a** Representative IMARIS 3D view of raw immunofluorescent Z-stack images: GFAP (red), NHE1 (green) and DAPI (blue), and 3D surface reconstruction of astrocytes based on GFAP immunostaining (red) in CC. White arrow: the position of boxed astrocytes in surface reconstruction in immunofluorescent Z-stack images. **b** Quantitative analysis of sum of soma volume, process volume, process mean diameter and terminal points of process for sham, BCAS+Veh, BCAS+HOE (i.p.) and BCAS+HOE (pump) groups. Data are presented as mean ± SD. *n* = 5–6/group. *p < 0.05, **p < 0.01, ***p < 0.001, ****p < 0.0001

Astrogliosis is a characteristic pathology in VCID and BCAS-induced white matter lesions [11, 12, 14]. We analyzed changes in GFAP^+^ astrocytes in the CC and EC white matter tracts from post-MRI ex vivo brains**.** Low numbers of GFAP^+^ cell counts were detected in sham brains (Fig. 3a–d), with basal levels of GFAP and NHE1 protein expression (arrow) in homeostatic astrocytes, which displayed small soma and fine processes (arrow, Fig. 3a, c). In contrast, the BCAS+Veh brains exhibited significantly higher GFAP intensity and increased GFAP^+^ cell counts in the CC and EC (*p* < 0.05, Fig. 3a–d). The IMARIS analysis of 3D structural changes of reactive astrocytes showed that compared to sham brains, GFAP^+^ astrocytes in the BCAS+Veh brains displayed significantly larger soma volume and process diameter in CC, along with more process terminal points, indicating hypertrophy of reactive astrocytes (arrows, *p* < 0.05, Fig. 4a, b). However, the 3D structures of reactive astrocytes in the BCAS+HOE brains exhibited astrocytic morphology similar to sham brains, with smaller soma volume and process diameter or less process terminal points. Similar patterns of morphologic changes of astrocytes in EC among these three groups were also detected (data not shown). In addition, we also detected an increased number of Iba1^+^ microglia, some of which showed amoeboid morphology in the CC and EC of the BCAS+Veh brains (**supplemental Fig. S****3****a, b**), and co-localization with NHE1 protein (**arrow**, **supplemental Fig. S****3****a**). Collectively, these data indicate that blocking NHE1 protein with HOE642 reduces both astrogliosis and microglial activation in white matter, which likely prevented BCAS-induced demyelination.

### Effects of NHE1 blockade on the BCAS-induced microstructural changes of hippocampus

**Fig. 5:**
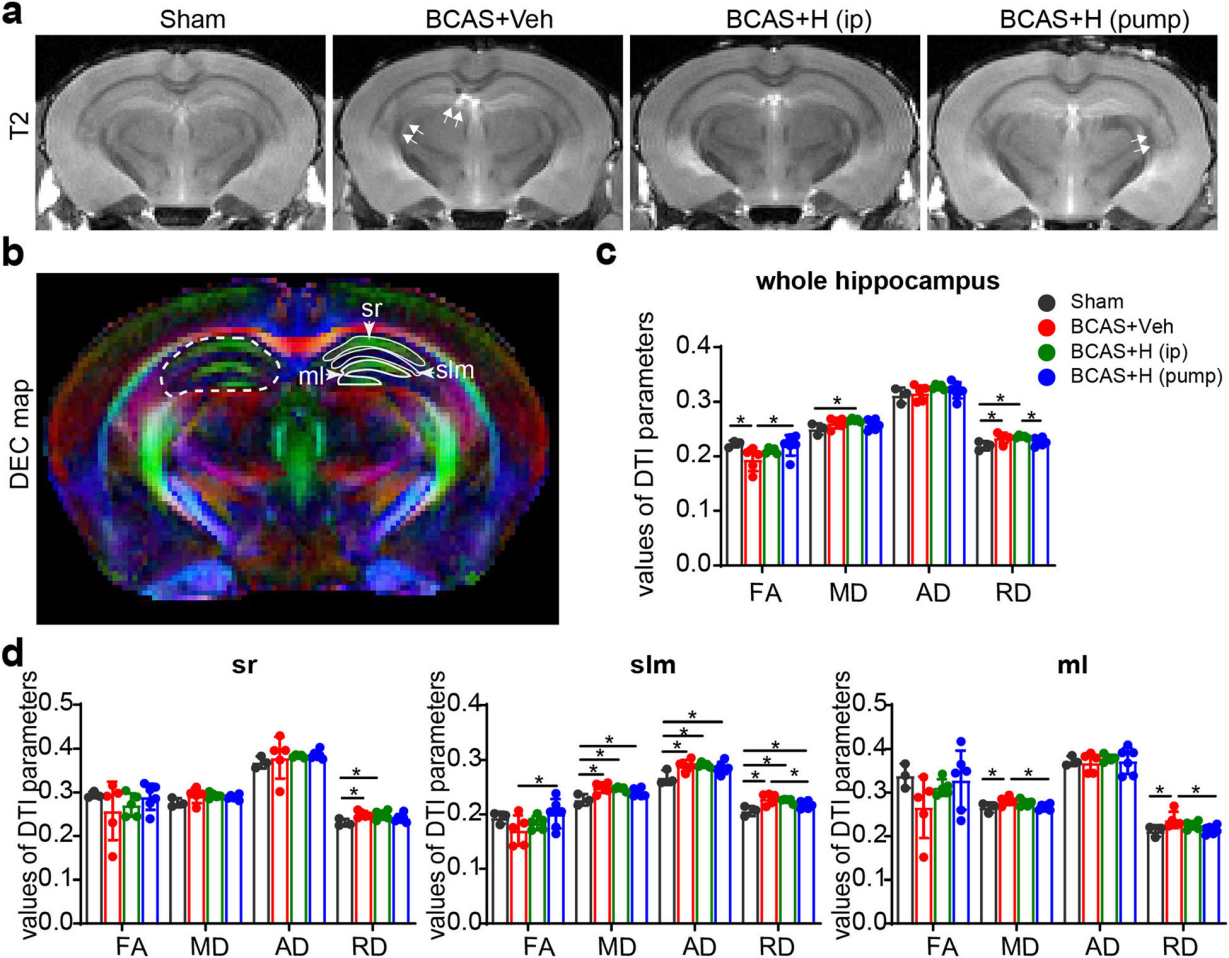
MRI revealed tolerance of HOE642-treated mice to BCAS-induced hippocampal injury. **a** Representative T2-weighted images of ex vivo brains of sham, BCAS+Veh, BCAS+HOE (i.p. or pump) mice at 30 days post-surgery. Double white arrow indicates damage on T2-weighted images. **b** Representative DEC maps of ex vivo brain showing ROIs in whole hippocampus (white dash line), stratum radiatum (sr), stratum lacunosum-meloculare (slm), and molecular layer (ml). **c** Quantitative analysis of FA, MD, AD, RD values of whole hippocampus. **d** Quantitative analysis of FA, MD, AD and RD values of different hippocampal regions. Data are presented as mean ± SD. *n* = 3–6/group. *p < 0.05

**Fig. 6:**
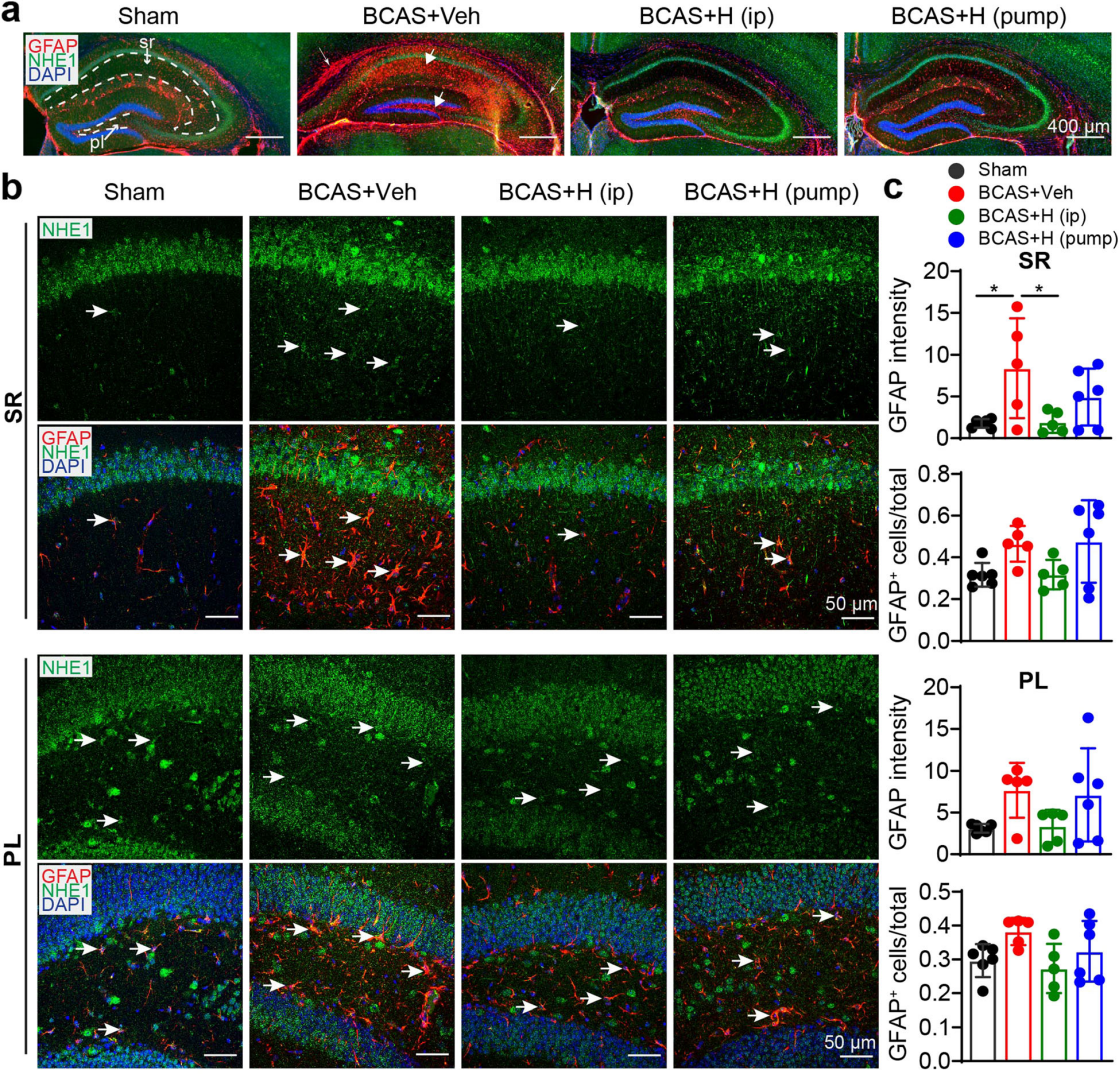
Inhibition of NHE1 protein with its inhibitor HOE642 in BCAS mice attenuated hippocampal astrogliosis. **a** Representative overview of NHE1 protein (green) and GFAP protein (red) expression in hippocampus at 30 days post-surgery. Thin arrow: increased GFAP^+^ astrocytes in CC and EC. Thick arrow: reactive GFAP^+^ astrocytes in hippocampus. Magnification ×10, scale bar = 400 μm. **b** Representative images of NHE1 (green) and GFAP (red) staining in stratum radiatum (SR) under CA1 field of hippocampus and in polymorph layer (PL) of dentate gyrus at 30 days post-surgery. Magnification ×40, scale bar = 50 μm. **c** Quantitative analysis of GFAP^+^ cells. Data are presented as mean ± SD. *n* = 5–6/group. *p < 0.05

As the Y maze spontaneous alternation rate indicates for spatial working memory, which requires hippocampal integrity [33], we examined whether HOE642 treatment affected BCAS-induced damage in the hippocampus. No significant NeuN^+^ neuron loss (**supplemental Fig S****4**) or changes in DTI parameters in the sensorimotor cortices were detected among the four experimental groups (data not shown). Ex-vivo T2-weighted MRI showed slight injury to the hippocampus of the BCAS+Veh and BCAS+HOE (pump) mice (double arrow, Fig. 5a). In contrast, DTI metrics measurement revealed that the whole hippocampus (from bregma − 0.94 to − 4.04 mm in both hemispheres) of the BCAS+Veh mice exhibited significantly lower FA values but higher RD values, and a trend of higher MD values, compared to sham brains (*p* < 0.05, Fig. 5b, c). The reduced FA along with increased MD and RD have been broadly reported to be associated with demyelination [34]. In further analysis of different hippocampal regions, the BCAS+Veh brains displayed higher MD and RD values in the stratum lacunosum moleculare (slm) and molecular layer (ml) of the dentate gyrus (*p* < 0.05, Fig. 5b–d). Interestingly, the BCAS+HOE (pump) mice exhibited values of DTI metrics (either in the whole hippocampus or its strata) that are comparable with sham mice (p > 0.05, Fig. 5b–d). But, the BCAS+HOE (i.p.) group showed similar DTI metrics (FA, RD and MD values) with the BCAS+Veh mice. In assessing hippocampal gliosis in these brains with immunostaining of GFAP^+^ reactive astrocytes, we detected substantial reactive astrogliosis in the hippocampus of the BCAS+Veh brains (arrow, Fig. 6a), with a significant increase in GFAP intensity in the stratum radiatum (sr) of the hippocampus and an increase in GFAP^+^ astrocytes, albeit insignificant, in polymorphic layer (pl) of the dentate gyrus (arrow, Fig. 6b, c). In contrast, the BCAS+HOE (i.p.) group exhibited attenuated reactive GFAP^**+**^ astrocytes in sr compared with the BCAS+Veh mice (*p* < 0.05, Fig. 6b, c), but this change was not detected in the sr of the hippocampus in the BCAS+HOE (pump) mice (Fig. 6b, c). Additionally, the BCAS+HOE-treated brains (i.p. or pump) also exhibited the absence of activation of microglia in the sr of the hippocampus (Supplemental Fig. S5a-c). Taken together, inhibition of NHE1 protein with HOE642 attenuated BCAS-induced gliosis in the hippocampus, which likely contributes to preserving hippocampal integrity and cognitive function. Considering the contributions of astrogliosis to increasing FA and decreasing RD values in DTI [35], the discrepancy in hippocampal astrogliosis and changes of DTI metrics in the BCAS+HOE-treated brains (i.p. or pump) is addressed in the “Discussion” section.

### Correlations of cognition function and structural integrity of white matter and hippocampus

**Fig. 7:**
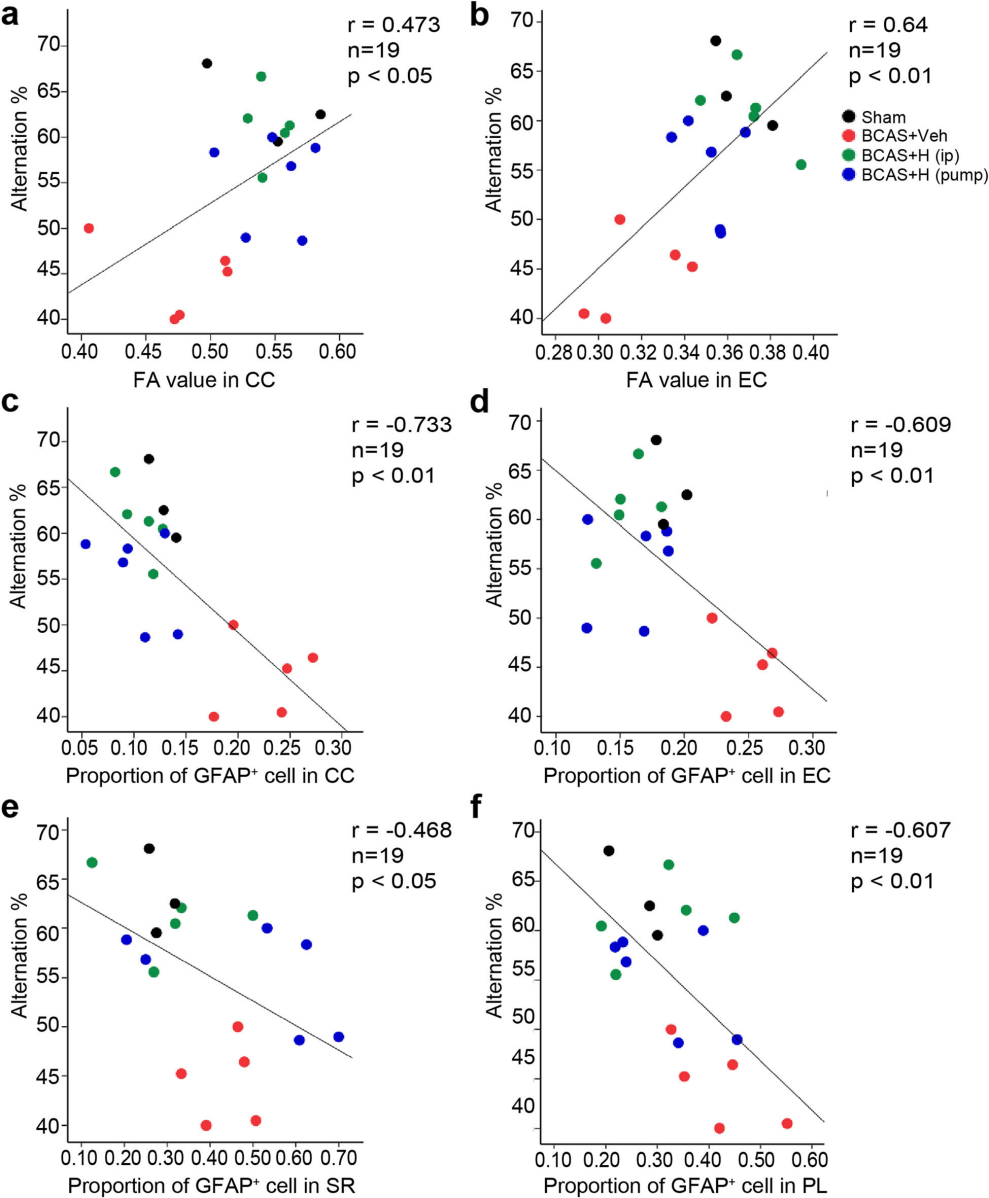
Correlation between alternation and microstructural change and astrogliosis in white matter tracts and hippocampus. **a** Pearson correlation between alternation in Y maze test and FA value in corpus colusum (CC). **b** Pearson correlation between alternation and FA value in external capsule (EC). **c** Pearson correlation between alternation and GFAP^+^ cell counts in CC. **d** Pearson correlation between alternation and GFAP^+^ cell counts in EC. **e** Pearson correlation between alternation and GFAP^+^ cell counts in hippocampal stratum radiatum (SR). **f** Pearson correlation between alternation and GFAP^+^ cell counts in poly morph layer (PL) of dentate gyrus. *n* = 19

We conducted Pearson’s correlation analysis to explore the relationships between BCAS-induced gliosis, white matter or hippocampal structural damage, and mouse cognitive function deficits. We found that the alternation rate of mice in Y maze test was correlated with FA value change in CC (*r* = 0.473, *p* < 0.05) and EC (*r* = 0.64, *p* < 0.05) (Fig. 7a, b and supplemental Table S1). At the cellular level, elevation of reactive GFAP^+^ astrocytes in CC (*r* = − 0.733) or EC (*r* = − 0.609) and activated Iba1^+^ microglia in CC (*r* = − 0.622) or EC (*r* = − 0.653) were negatively correlated with spatial working memory of mice in Y maze test (Fig. 7c, d and supplemental Table S1). In the hippocampus, GFAP^+^ astrocyte counts and Iba1^+^ counts in the sr or pl of the hippocampus significantly correlated with the alternation rate in Y maze test (Fig. 7e, f and supplemental Table S1). The alternation rate in Y maze task was also significantly correlated with RD in slm and ml of hippocampus (*r* = 0.55–0.6, supplemental Table S1), although we did not detect a correlation between cognitive function and DTI metrics of the whole hippocampus. Taken together, these findings suggest that glial activation in white matter tracts and hippocampus is closely associated with spatial working memory impairment induced by BCAS. Pharmacological inhibition of NHE1 protein attenuates glial activation, which led to the preservation of white matter integrity and hippocampal integrity and improvement of spatial working memory.

### Pharmacological inhibition of NHE1 protein revealed reduced transcriptome profiles of ROS production and inflammatory responses in astrocytes

**Fig. 8:**
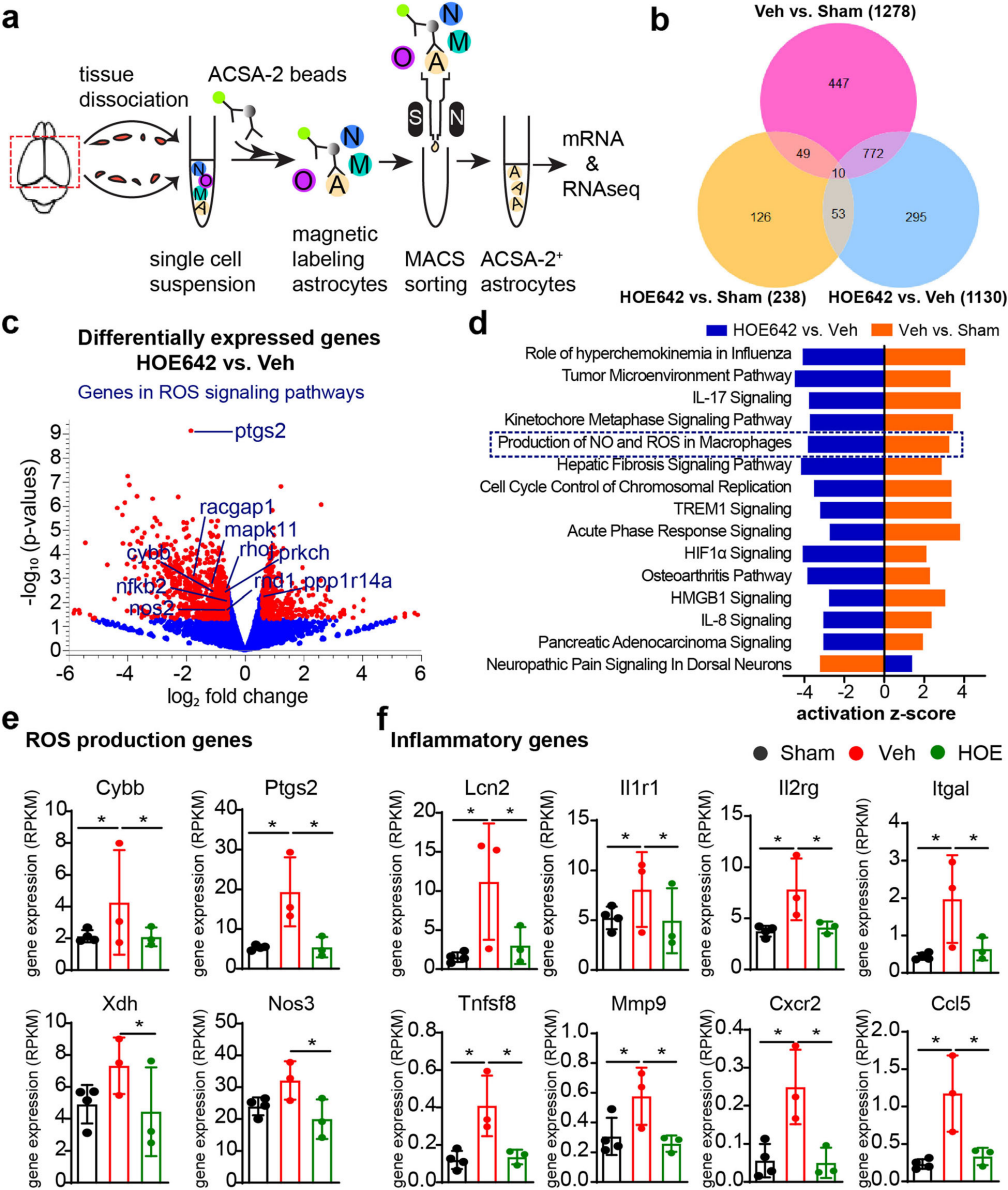
Bulk RNA-seq analysis of transcriptome changes in brain astrocytes. **a** Schematics of brain astrocyte isolation for bulk RNA-seq analysis from sham, BCAS+Veh (i.p.), and BCAS+HOE642 (i.p.) mice at 30 days post-surgery. *n* = 4/group. **b** Venn diagram depicting differential gene expression in brain astrocytes (*p* value ≤ 0.05 and FC≥1.5). **c** Volcano plots illustrate the gene expression pattern detected with *p* value ≤ 0.05 and FC≥1.5. Red dot: significant differentially expressed gene; Blue dot: non-significant differentially expressed gene. **d** Comparison analysis of significantly altered canonical pathways by ingenuity pathway analysis (IPA) between BCAS+Veh vs. sham group and BCAS+HOE vs. BCAS+Veh group. *p* value < 0.05 and *Z*-score ≥ 3. **e** ROS production genes expression. Data are RPKM values shown as mean ± SD, *n* = 3-4. *p ≤ 0.05 and FC >1.5. **f** Inflammatory gene expression. Data are RPKM values shown as mean ± SD, *n* = 3–4. *p ≤ 0.05 and FC > 1.5

To understand the underlying mechanisms of NHE1 protein in astrogliosis in BCAS brains, we conducted an RNA-seq of MACS-isolated brain astrocytes from sham, BCAS+Veh (i.p.), and BCAS+HOE (i.p.) mice (Fig. 8a). The purity of the isolated ASCA2^+^ astrocytes was validated by their highly expressed gene markers *Atp1b2*, *Slc2a1*, *Aqp4*, *Gfap*, *Aldh1l1* that are specifically enriched in astrocytes (Supplemental Fig. S6). Specifically, no marker genes for neurons and only trace levels of specific marker genes for microglia, oligodendrocytes, or endothelial cells were detected. The bulk RNA-seq analysis identified 1130 differentially expressed genes in the BCAS+HOE-treated brain astrocytes, compared with the BCAS+Veh-treated brain astrocytes (Fig. 8b), including 340 upregulated genes and 790 downregulated genes (Fig. 8c). GO analysis using DAVID software showed that the upregulated genes were involved in neuroprotective biological processes, such as “signal transduction,” “cell adhesion,” “multicellular organism development,” “nervous system development,” “myelination,” and “ion transport” (*p* < 0.05) (Supplemental Fig. S7), whereas the downregulated genes in the BCAS+HOE-treated brain astrocytes were mainly involved in biological processes of “cell cycle” and “inflammatory responses” (Supplemental Fig. S8).

In light of diverse transcriptomic profiling of reactive astrocytes in different disease models [36, 37], we further explored the changes of reactive astrocyte transcriptomes in our study. In comparison to the sham controls, the BCAS+Veh astrocytes showed significant upregulation of classic transcriptomic signatures of reactive astrocytes, including pan-reactive signatures (*Vim*, *Lcn2*, *Cd44*, *Steap4*, *Cxcl10*, *Gfap*, *Serpina3n*, *Osmr*, *Timp1*), the reported clustered A1 phenotype genes (*Fkbp5*, *Gbp2*, *H2-D1*, *H2-T23*, *Iigp1*, *C3*, *C4b*, *Psmb8*, *Serping1*, *Srgn*) or A2 phenotype genes (*Cd14*, *Emp1*, *Ptgs2*, *Tgm1*) (*p* ≤ 0.05 and FC ≥ 1.5, Supplemental Fig. S9). In contrast, the astrocytes isolated from the BCAS+HOE-treated mice presented transcriptome profiles that resembled the sham control astrocyte profile, e.g., with downregulated pan-reactive signatures (*Vim*, *Lcn2*, *Cd44*, *Cxcl10*, *Hspb1*, *Gfap*, *Serpina3n*, *Osmr*, *Timp1*), and lower expression level of the clustered genes (*Gbp2*, *H2-D1*, *C3*, *C4b*, *Psmb8*, *Serping1*, *Srgn*) and (*Cd14*, *Emp1*, *Ptgs2*, *Tgm1*) (supplemental Fig. S9). Collectively, these results demonstrated that BCAS triggered proinflammatory transcriptome changes of reactive astrocytes, and pharmacological inhibition of NHE1 prevented such changes in astrocytes.

In addition, the pathway enrichment analysis using IPA for DEGs reveals that BCAS induced activation of the inflammation and cell cycle-related pathways, compared with sham groups, and that HOE642 treatment inhibited these changes **(***p* < 0.05 and *Z*-score ≥ 3, Fig. 8d). Of note, the “production of NO and ROS in macrophages” pathway was significantly elevated by BCAS while suppressed by HOE treatment (Fig. 8d). The gene *Cybb* encoding NOX2 subunit gp91^phox^ was upregulated in astrocytes of the BCAS+Veh mice but was significantly downregulated in the BCAS+HOE mice **(***p* < 0.05 and FC > 1.5, Fig. 8c, e). In addition, BCAS triggered significant elevation of the expression of other reactive oxygen species (ROS) signaling related genes, including NOX2 subunit rac-related genes (*Racgap1*, *Rhoj*, *Rnd1*), Pro-inflammatory and ROS production enzyme cyclooxygenase-2 (COX2) gene (*Ptgs2*), ROS signaling downstream genes (*Mapk11*), and inflammatory genes (*Lcn2*, *Il1r1*, *Il2rg*, *Tnfsf8*, *Mmp9*, *Cxcr2*, *Ccl5*, *Itgal*, etc.). In contrast, HOE642 treatment prevented upregulation of these signature genes **(***p* ≤ 0.05 and FC > 1.5, Fig. 8 c, e, and f and supplemental Fig. S10). In addition, HOE642 treatment significantly downregulated the NOX2 activator protein kinase C gene (*Prkch*), other ROS production genes (*Nos2*, *Nos3*, *Xdh*), and ROS signaling downstream transcription factor gene (*Nfkb2)* (*p* ≤ 0.05 and FC > 1.5, Fig. 8c, e, and f and supplemental Fig. S10). Taken together, the bioinformatic analysis of astrocytes revealed that inhibition of NHE1 protein with HOE642 prevented BCAS-induced astrocytic transcriptome changes for ROS and inflammatory cytokines release, which collectively drive attenuation of gliosis, demyelination, and cognitive dysfunction.

### NHE-1 blockade attenuated astrocytic NOX2 activation in white matter

**Fig. 9:**
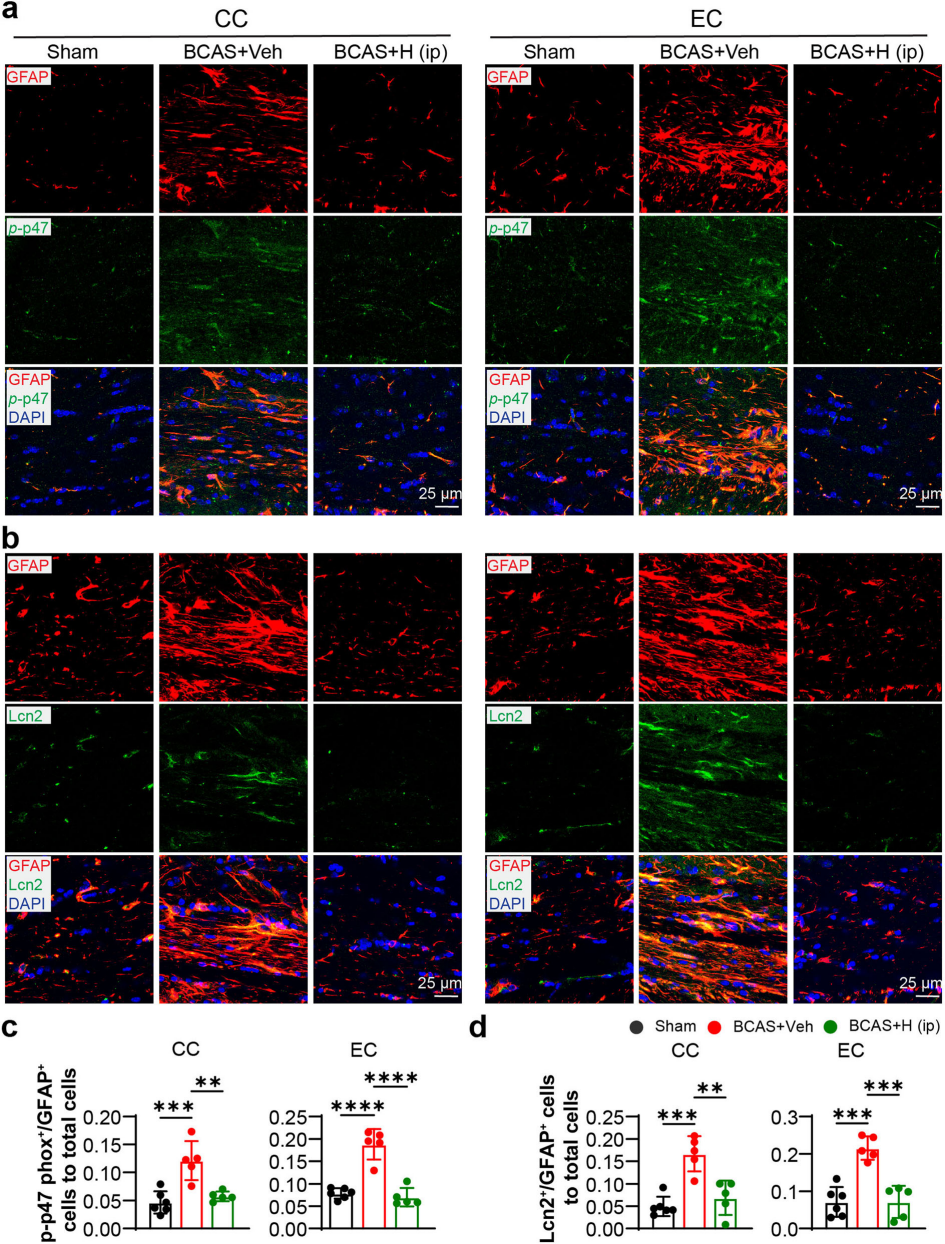
HOE642 treatment attenuated astrocytic NOX2 activation and LCN2 expression in white matter. **a** Representative staining images of phosphorylated p47 phox (green) and GFAP (red) in CC and EC at 30 days post-surgery. Magnification ×40, Scale bar = 25 μm. **b** Representative immunostaining images of LCN2 (green) and GFAP (red) in CC and EC. Magnification ×40, scale bar = 25 μm. **c** Quantitative analysis of the co-labeled p-p47^+^/GFAP^+^ cells in CC and EC. **d** Quantitative analysis of the co-labeled LCN2^+^/GFAP^+^ cells in CC and EC. All data are presented as mean ± SD. *n* = 5–6. **p < 0.01, ***p < 0.001, ****p < 0.0001

NHE1-mediated H^+^ extrusion promotes NOX2 activation in microglia and neurons [21, 38]. To further investigate that blockade of NHE1 protein reduced the ROS production and pro-inflammation resulting from attenuating NOX2 activity in white matter reactive astrocytes, we examined changes of expression of phosphorylated cytosolic subunit p47 (*p*-p47 phox) of NOX2 in astrocytes in CC and EC white matter tracts, since phosphorylation of cytosolic subunits p40, p47, and p67 is required for NOX2 activation [39]. We also probed changes of LCN2 (Lipocalin 2) protein expression since it stimulates the classical proinflammatory reaction in astrocytes [40]. As shown in Fig. 9, compared with the sham group, the BCAS+Veh mice showed abundant expression of phosphorylated p47 phox (*p*-p47) and LCN2 in astrocytes in CC and EC white matter tracts. The BCAS+HOE642 (i.p.) treatment suppressed phosphorylation of p47 phox and LCN2 protein upregulation in astrocytes (*p* < 0.05, Fig. 9). Collectively, these results suggested the blockade of NHE1 protein inhibited ROS production and pro-inflammatory activation in astrocytes, which contributed to the attenuation of gliosis, demyelination, and cognitive dysfunction.

## Discussion

### BCAS-induced astrogliosis in the white matter and hippocampus and cognitive function deficits

CCH causes white matter lesions and hippocampal atrophy and is one of the major contributions to VCID [41, 42]. Among various VCID models, the BCAS model induces mild chronic hypoperfusion and simulates key characteristic pathogenesis of VCID, including white matter lesions, gliosis, inflammation [3]. BCAS triggers reactive astrogliosis in the white matter [32, 43], cortex, striatum, and hippocampus [44]. In our study, we detected substantial activation of glial cells (GFAP^+^ reactive astrocytes and Iba1^+^ microglia) in the CC, EC, and hippocampus at 30 days post-BCAS, which were associated with white matter and hippocampal demyelination and microstructural damage. Importantly, chronic administration of NHE1 inhibitor HOE642 in BCAS mice significantly reduced cell counts of GFAP^+^ reactive astrocytes and Iba1^+^ microglia in white matter as well as in the hippocampus. This reveals the therapeutic potential of targeting the astrocytic NHE1 protein for reducing astrogliosis in VCID.

### Correlation of brain microstructural damage, astroglial activation, and cognitive function impairment

The strength of our study is that we completed cognitive behavioral tests, white matter microstructural analysis with MRI DTI, and evaluation of pathologic cellular changes with immunofluorescence staining in the same cohorts of mice, which allowed us to perform correlation analyses. Decreased FA is highly sensitive to detect white matter microstructural damage, and increased RD and decreased AD values are indicative of demyelination and axonal degeneration, respectively [34, 35]. We observed a linear correlation between the number of GFAP^+^ reactive astrocytes and FA values in white matter (CC and EC), which is consistent with reports for a detrimental role of astrogliosis in white matter lesions [32, 45]. However, in addition to MRI DTI and immunostaining analysis to assess the correlation of changes in MBP and astrocytic GFAP protein in white matter tissues, future Western blot quantification could further strengthen our findings. In addition, the FA values in white matter linearly correlated with the alternation performance in the Y maze test in mice, which suggests chronic hypoperfusion injury-mediated astrogliosis contributes to white matter lesions and ensuing spatial working memory deficit. We also detected a significant correlation between the number of reactive astrocytes in the hippocampus and the alternation performance in the Y maze test. Two treatment regimens with the selective NHE1 inhibitor HOE642 (cariporide) (daily bolus i.p. injection or continuous pump delivery for over 28 days) improved spatial working memory, likely due to inhibition of reactive astrogliosis in white matter and hippocampus. Blood-brain barrier (BBB) damage was detected as early as 1 day post BCAS surgery in mice [3, 46], which can facilitate HOE642 penetration into the BCAS parenchymal brain tissues. It is also reported that intravenous administration of HOE642 in rats is neuroprotective in permanent ischemic stroke model [47], another evidence for HOE642 penetration in ischemic brains with the compromised BBB.

However, some discrepancy in the correlative analysis of hippocampal astrogliosis and demyelination was detected in the HOE642 treatment (i.p.) mice, in which no improved hippocampal DTI indices (i.e. increased FA or decreased RD) were observed. The reasons for this uncoupling are not apparent. Studies have reported that increased reactive astrogliosis could also elevate FA and decrease RD in DTI metrics due to their coherent arrangement or decreased the inter-space between axons resulting from axon swelling and astrocytic hypertrophy, which mask demyelination [35, 48]. The lack of changes in the DTI metric values in the hippocampus of the BCAS+HOE (i.p.) mice could be due to the removal of the confounding influences of astrogliosis.

### Pharmacological inhibition of NHE1 protein reduces astrocytic proinflammatory transcriptome changes and NOX2 complex activation

The underlying mechanisms for NHE1 in glial activation and inflammation are not completely understood. Our RNA-seq analysis of isolated astrocytes revealed that inhibition of NHE1 with HOE642 treatment substantially downregulated gene expressions involved cell cycle and inflammation responses to the BCAS-induced cerebral hypoperfusion. NHE1 blockade inhibited the gene expression of NOX2 (*Cybb*) and its subunit rac1/2 activity-related genes (*Racgap1*, *Rhoj*, *and Rnd1*), which are involved in the production of ROS [49]. Moreover, in astrocytes of the BCAS+Veh mice, *Prkch* gene encoding protein kinase C (PKC), a key activator of NOX2 by phosphorylation of NOX2 subunits [39], was significantly upregulated, but the gene of PKC inhibitor (*Ppp1r14a*) was significantly downregulated. Interestingly, HOE642 treatment blocked the *Prkch* upregulation and *Ppp1r14a* downregulation, which could synergistically inhibit the phosphorylation of NOX2 subunits (p40, p47, p67). Our immunostaining data also showed that NHE1 inhibition attenuated NOX2 activity (reflected with reduced phosphorylation of the p47 subunit). In addition, the expression of the COX2 gene (*Ptgst2*), another important source of ROS [50] and canonical target of anti-chronic inflammation [51], was significantly reduced after the NHE1 inhibition. Inflammatory related gene product such as astrocytic LCN2 (*Lcn2*) plays a pivotal role in gliosis, recruitment of macrophages, and release of pro-inflammatory cytokines [40]. Blocking NHE1 activity significantly attenuated LCN2 protein and mRNA expression in astrocytes.

The molecular underlying mechanisms are not known. We have previously reported that NHE1 protein-mediated H^+^ extrusion in microglial cells prevents intracellular acidosis and is coupled with microglial activation and pro-inflammatory cytokine release via sustaining NOX2 function [19, 21]. It has also been reported that NHE inhibitors (amiloride and EIPA) have anti-inflammatory effects by decreasing LPS-induced COX2 expression and production of PGE2 in the murine macrophage cells [51], which may result from the alkalinization of extracellular environment [52]. Future studies are needed to determine whether NHE1-mediated regulation of pH_i_ homeostasis in astrocytes directly modulates transcriptomes of ROS production and inflammation, and has an impact on ROS and cytokine production.

Pathological glial activation has a destructive effect on white matter integrity through their pro-inflammatory effects [53, 54]. Reactive astrocytes showed significantly increased pro-inflammatory transcription factor NF-κB activation and transgenic inhibition of astroglial NF-κB signaling preserved white matter integrity and improved cognitive function in BCAS mice [55]. Reactive astrocytes lost the ability to facilitate oligodendrocyte lineage cell maturation or developed abnormal glutamate metabolism [32, 56]. Moreover, there is close crosstalk between astrocytes and microglia in response to pathological stimuli [57]. Considering that the detrimental ROS and nitric oxide (NO) from reactive astrocytes can in turn to promote the activation of microglia [57, 58], the effects of pharmacological blockade of NHE1 protein in attenuating microglial activation could be indirect through reducing reactive astrogliosis and/or via directly inhibiting microglial NHE1 activity, the latter was shown to be involved in stroke-mediated proinflammatory microglial polarization [17].

A1, A2, and pan-reactive astrocyte transcriptomic signature analysis has been widely reported [37, 59]. We detected upregulation of genes in BCAS+Veh astrocytes, such as *Lcn2*, *Serpina3n*, *Osmr*, *C3*, *C4b*, which are well-known pro-inflammatory markers of reactive astrocytes [36, 37]. These cells did not show changes in top A2 phenotype genes *S100A10* or *Ptx3* but displayed elevation of other A2 signature genes including *Cd14*, *Ptgs2* that are involved in inflammatory responses. Importantly, our results demonstrated that BCAS triggered reactive astrocyte response with upregulation of a range of inflammation-related genes (*Lcn2*, *Serpina3n*, *Osmr*, *C3*, *C4b*, *Cd14*, *Ptgs2*, etc.), while NHE1 inhibitor HOE642 treatment suppressed these gene transcripts. These analyses indicate that the BCAS triggered distinctive phenotypes of reactive astrocyte transformation, which does not fall in the binary A1/A2 polarization, as reported recently [60].

### Protective effects of administration of NHE1 inhibitors in vivo

Protective effects of pharmacological NHE1 inhibition have been reported in various brain injury studies. In mouse neonatal hypoxia-ischemia brain injury models, administration of NHE1 inhibitor HOE 642 (0.5 mg/kg, i.p.) in neonatal mice at 10 min, 24 h, and 48 h after hypoxia reduced CC damage as well as hippocampal neurodegeneration [23, 61]. In the mouse model of ischemic stroke, HOE 642 (0.5 mg/kg/d, i.p.) given at 0–7 days post-stroke significantly decreased infarct volume, microglial activation, and pro-inflammatory stimulation, but did not attenuate reactive astrocytes in the peri-infarct area [19]. Moreover, in a mouse global cerebral ischemia model by bilateral common carotid artery occlusion, administration of another NHE1 inhibitor amiloride (10 mg/kg, i.p.) after ischemia induction, significantly reduced neuronal loss, gliosis, and oxidative damage in the hippocampus [62]. Lastly, in a rat spontaneous subarachnoid hemorrhage model, intravenous injection of HOE642 (15 mg/kg) at 20 min before induction of subarachnoid hemorrhage decreased inflammatory reactions, oxidative stress, and neuronal loss [63]. In all these prior studies, NHE1 inhibitors were administered in the acute post-injury phase, which lasted no longer than one week. In contrast, in our study, HOE642 (0.3mk/kg/day), administered for nearly 30 days, exhibited protective effects with improved cognitive functions and reduced brain pathology. These are encouraging new findings about the therapeutic potential for chronic NHE1 inhibition for the CCH-mediated brain damage.

However, the osmotic pump administration of HOE642 in our study was less effective with the underlying mechanisms still unknown. It could be due to HOE642 instability in the saline solution [64] in the osmotic pump or cumulative toxicity resulting from higher HOE642 plasma concentrations. Studies in a cardiac disease model have reported that the chronic administration of HOE642 (30mg/kg/day) in rat drinking water for 1 month [65] or at 6000 ppm in mouse chow for 8 months [66] produced cardiac protective effects, without reported adverse effects. Considering the half-life of HOE642 at ~ 3.5 h in human serum and 1.5 h in rat serum [67, 68], there is a possibility that the osmotic pump with a constant delivery of HOE642 could accumulate higher serum concentrations and result in toxicity as indicated in the study by Kisker et al, that more than 5-fold increase in the area under the concentration time curve was detected in the osmotic pump delivery than in i.p. injection [69]. Future studies are needed to investigate the pharmacokinetics of HOE642 via different administration routes to determine the optimal dosing regimen. Additionally, the sample sizes in our study are relatively small; despite that, we were able to determine the statistical significance for neurological deficit assessment, immunostaining, and qRT-PCR and RNA-seq analyses. Determination of the potential therapeutic benefits of NHE1 pharmacological inhibition in the CCH model is warranted with larger sample sizes.

## Conclusions

Our study demonstrated that gliosis in white matter tracts and hippocampus is associated with white matter demyelination, microstructural damage of hippocampus, and spatial working memory deficits after chronic cerebral hypoperfusion injury induced by BCAS. Reactive GFAP^+^ astrocytes displayed an elevated transcriptome for ROS production, inflammation, and NOX2 activation. Our study revealed that inhibition of NHE1 protein reduces brain astrocytic transcriptomes for ROS production and inflammation, as shown in the schematic summary in Supplemental Figure S10. Pharmacological inhibition of NHE1 activity with HOE642 attenuated reactive astrocyte gliosis, preserved white matter and hippocampal integrity, and improved cognitive function. These new findings suggest NHE1 protein as a potential therapeutic target for astrogliosis and VCID.

## Supplementary Information

**Additional file 1.**

## Data Availability

Supporting data and information about used material are available from the corresponding author on reasonable request.

## Abbreviations

CCH: Chronic cerebral hypoperfusion
NHE1: Na^+^/H^+^ exchanger 1
BCAS: Bilateral carotid artery stenosis
MRI: Magnetic resonance imaging
DTI: Diffusion tensor imaging
DEC: Directionally encoded color
T2WI: T2-weighted images
I.P. injection: Intraperitoneal injection
MBP: Myelin basic protein
NF-200: Neurofilament-200
GFAP: Glial fibrillary acidic protein
NADPH: Nicotinamide adenine dinucleotide phosphate
VCID: Vascular contributions to cognitive impairment and dementia
AD: Alzheimer’s disease
WMLs: White matter lesions
NOX: NADPH oxidase
CBF: Cerebral blood flow
rCBF: Regional CBF
OF: Open field
CC: Corpus callosum
EC: External capsule
FA: Fractional anisotropy
AD: Axonal diffusivity
RD: Radial diffusivity
MD: Mean diffusivity
ROIs: Regions of interest
DEGs: Differentially expressed genes
IPA: Ingenuity pathway analysis
GO: Gene ontology
DAVID: Database for Annotation Visualization and Integrated Discovery
SD: Standard deviation
SR: Stratum radiatum
PL: Polymorphic layer
SLM: Stratum lacunosum moleculare
ML: Molecular layer
ROS: Reactive oxygen species
COX2: Cyclooxygenase-2
Lcn2: Lipocalin 2
Rt-qPCR: Reverse transcription quantitative real-time PCR
PKC: Protein kinase C
