# Supplementary material for: Attenuating vascular stenosis-induced astrogliosis preserves white matter integrity and cognitive function
Source: J Neuroinflammation. 2021 Aug 28;18:187. doi: 10.1186/s12974-021-02234-8 (PMC8403348; doi:10.1186/s12974-021-02234-8)
Supplement: Supplementary file 1 — Additional file 1. [file 12974_2021_2234_MOESM1_ESM.docx]

**Attenuating vascular stenosis-induced astrogliosis preserves white matter integrity and cognitive function**

Qian Liu ^1, 2, 3^; Mohammad Iqbal H. Bhuiyan^2, 3^; Ruijia Liu ^2, 3^; Shanshan Song ^2, 3^; Gulnaz Begum ^2, 3^; Cullen B. Young ^2, 3^; Lesley M. Foley ^4^; Fenghua Chen ^2^; T. Kevin Hitchens ^4, 5^; Guodong Cao ^2, 6^; Ansuman Chattopadhyay ^7^; Li He^1^*; Dandan Sun ^2, 3, 6^*

^1^ Department of Neurology, West China Hospital, Sichuan University, Chengdu, Sichuan, 610041, China

^2^ Department of Neurology, University of Pittsburgh, Pittsburgh, Pennsylvania, 15213, USA

^3^ Pittsburgh Institute for Neurodegenerative Disorders, University of Pittsburgh, Pittsburgh, Pennsylvania, 15213, USA

^4^ Animal Imaging Center, University of Pittsburgh, Pittsburgh, Pennsylvania, 15213, USA

^5^ Department of Neurobiology, University of Pittsburgh, Pittsburgh, Pennsylvania, 15213, USA

^6^ VA Pittsburgh Healthcare System, Geriatric Research Education and Clinical Center, Pittsburgh, Pennsylvania, 15240, USA

^7^ Molecular Biology-Information Service, Health Sciences Library System, University of Pittsburgh, Pittsburgh, Pennsylvania, 15261, USA

**Running title**: NHE1 protein in astrogliosis

*Corresponding authors:

**Dandan Sun, M.D., Ph.D.**

Department of Neurology

University of Pittsburgh Medical Center

7016 Biomedical Science Tower 3

3501 Fifth Ave.

Pittsburgh, PA 15260, USA

Phone number: (412) 624-0418

E-mail address: [sund@upmc.edu](mailto:sund@upmc.edu)

**Li He, M.D.**

Department of Neurology

West China Hospital, Sichuan University

No. 37, Wainan Guoxue Xiang

Chengdu, Sichuan, 610041, China

Phone number: (+86) 18980601670

Email address: heli2003new@126.com

**METHODS AND MATERIALS**

**Materials**

HOE-642 (Cariporide, #SML1360) was from Millipore Corporation (Burlington, MA). DAPI (4,6-diamino-2-phenylindole, dhydrochloride) was from Life Technologies Corporation (Carlsbad, CA). Rabbit anti-MBP antibody (ab40390) and rat anti-Lcn2 (ab70287) were from Abcam plc (Cambridge, UK). Rabbit anti-NHE-1 antibody (sc-28758) were from Santa Cruz biotechnology (Santa Cruz, CA). Mouse anti-GFAP antibody (3670s) was from Cell Signaling Technology (Danvers, MA). Rabbit anti-Neurofilament 200 (N4142) and mouse anti-NeuN antibody (MAB377) were from Millipore Corporation (Billerica, MA). Rabbit anti-Phospho-p47phox Antibody (PA536773) was from Invitrogen. Goat anti Iba1 (NB100-1028) was from Novus Biologicals (Centennial, CO). Goat-anti-rabbit AlexaFluor 488 (A11008), goat-anti-rabbit AlexaFluor 546 (A11035) or goat-anti-mouse AlexaFluor 546 (A11030) were from Life Technologies (Grand Island, NY). Donkey anti goat 488 (A-11055) and Donkey anti rabbit 546 (A10040) were from Thermo Fisher Scientific. Adult Brain Dissociation Kit (Miltenyi Biotec, Germany). RNeasy Micro Kit (Qiagen, 74004).


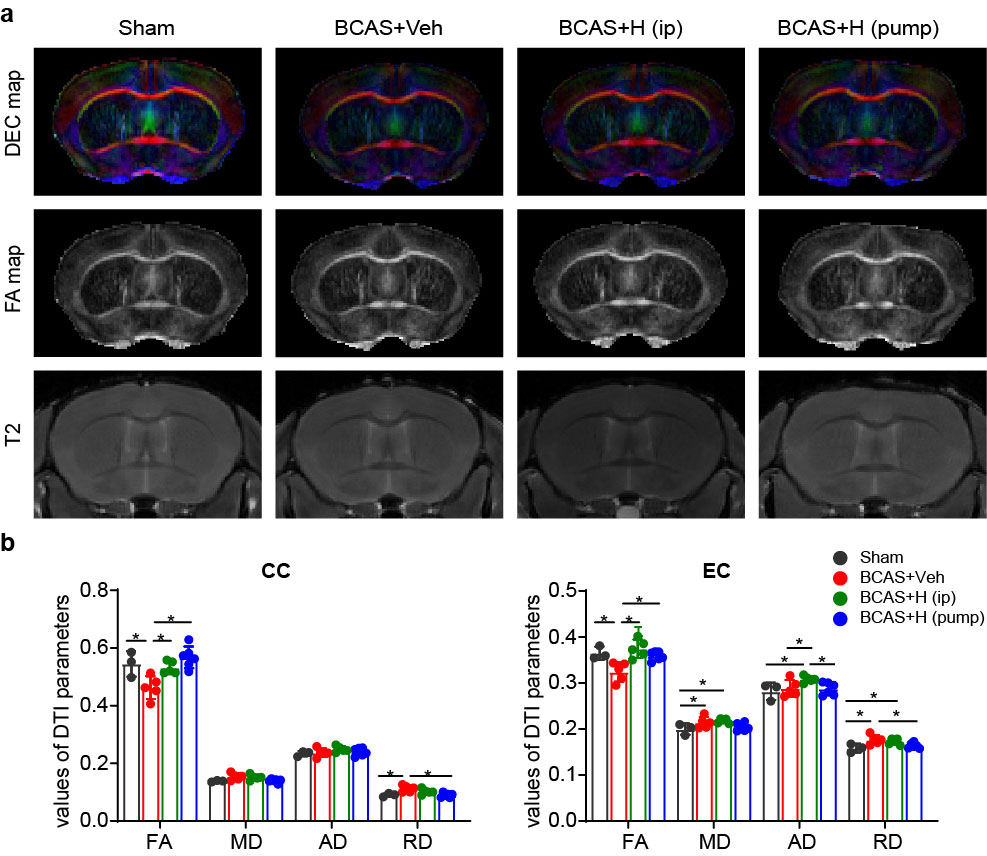


**Figure S1.** (**a)** Representative DEC, FA, T2 maps of white matter of ex vivo brains from Sham, BCAS+Veh (i.p.), BCAS+HOE (i.p.), or BCAS+HOE (pump) mice. (**b)** Mean values of DTI parameters in CC and EC of these brains. All data are presented as mean ± SD, n=3-6. *P < 0.05.

**
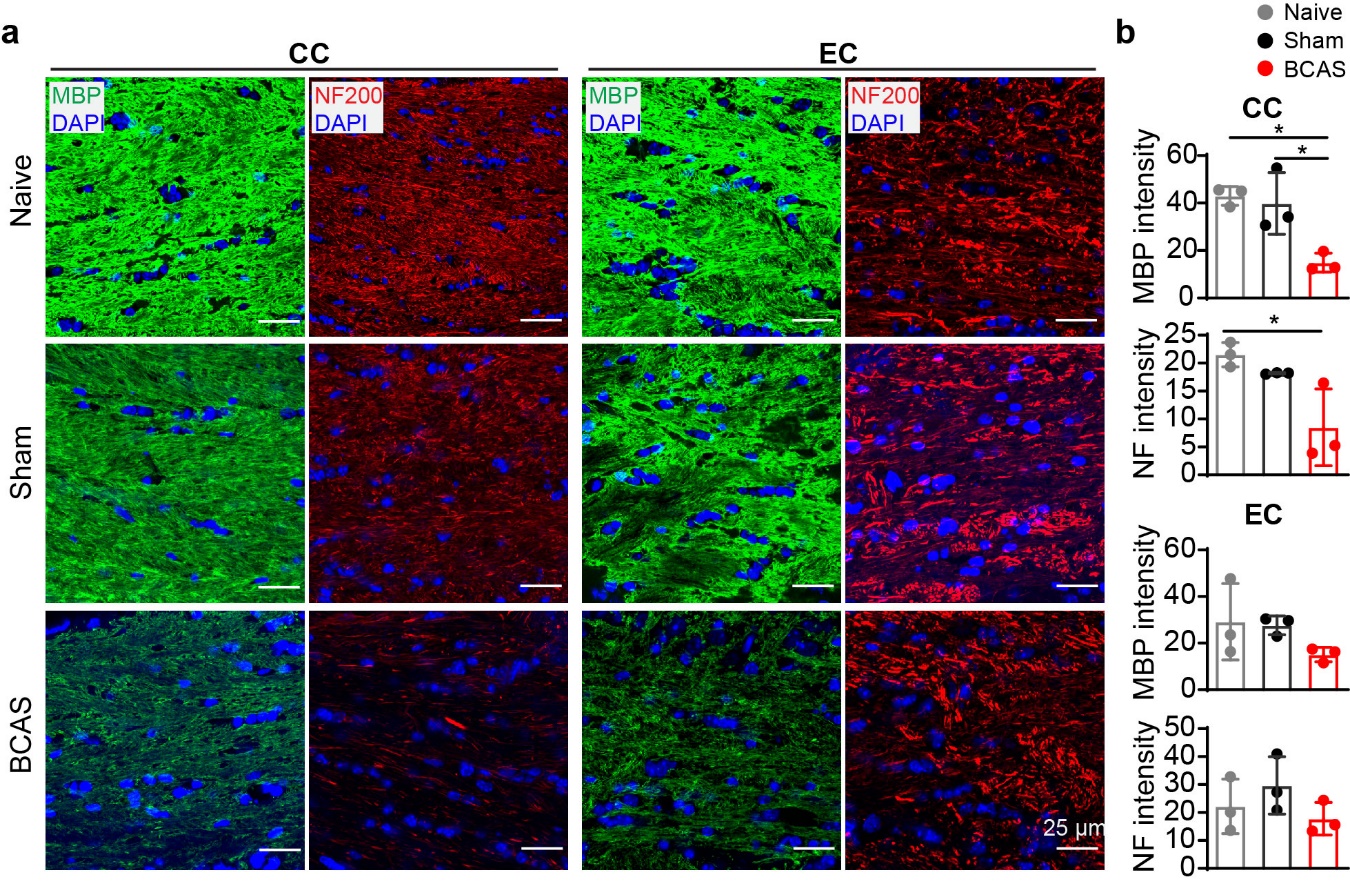
**

**Figure S2.** **BCAS-induced demyelination and neurofilament loss in white matter at 30 days post-surgery.** (a) Representative MBP (green) or NF200 (red) immunostaining in CC and EC. Scale bar=25 µm. (b) Quantitative analysis of the mean intensity of MBP or NF200 in CC and EC. All data are presented as mean ± SD. n=3. *P < 0.05.


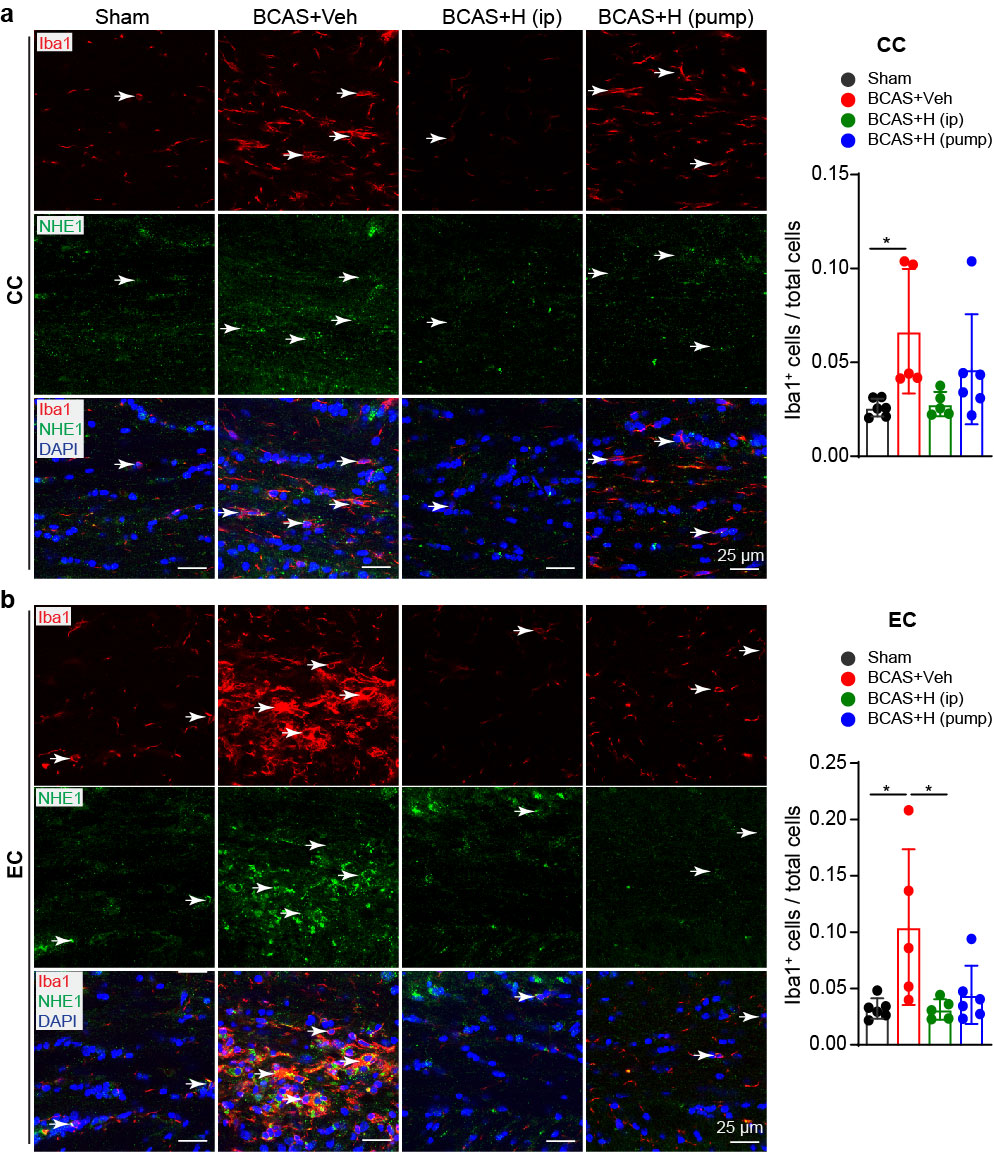


**Figure S3. HOE642 treatment suppressed BCAS-induced elevation of Iba1 microglia in white matter.** (a, b) Representative images of NHE1 expression in Iba1^+^ cells and quantitative analysis of Iba1^+^ cell counts in CC (a) and EC (b) of sham, BCAS+Veh (i.p.) and BCAS+HOE (i.p.) and BCAS+HOE (pump) mice. White arrow: Iba1^+^ microglia with NHE1 expression. Scale bar=25 µm. All data are presented as mean ± SD. n = 5-6. *P < 0.05.


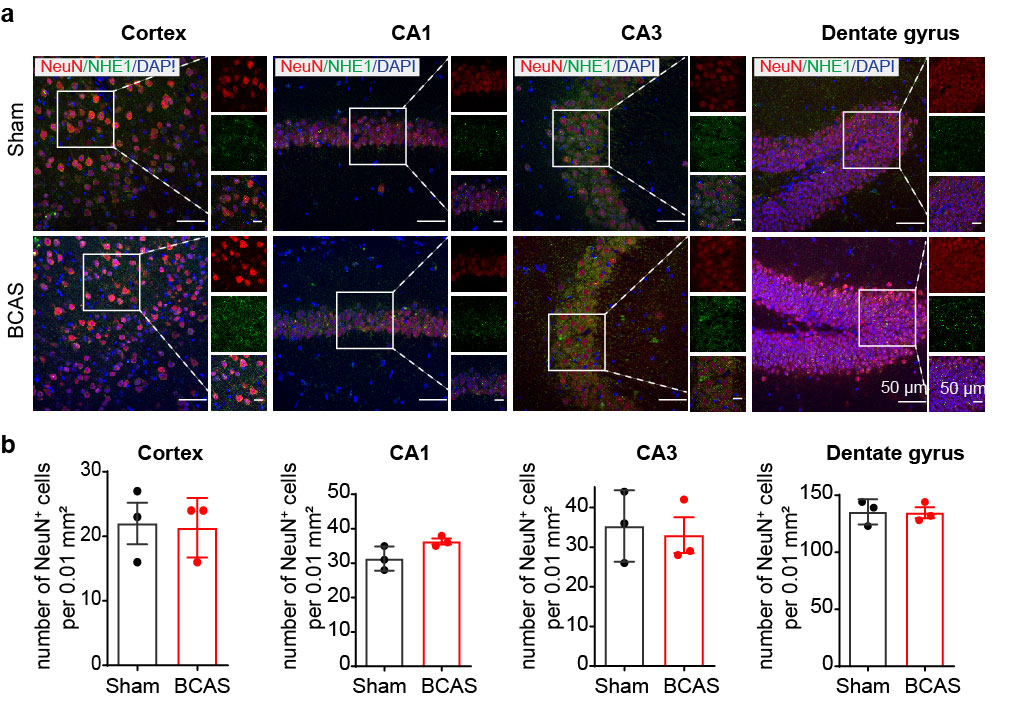


**Figure S4. Lack of BCAS-induced neuronal loss in cortex and hippocampus.** (a) Representative immunostaining images of NHE1 (green) and NeuN (red) in cortex, hippocampal CA1, CA3 and dentate gyrus in Sham and BCAS at 30d post-surgery. Selective detailed signal information (from the white square frames) in each channel was further displayed in the right panel. Scale bar=50 µm. (b) Quantitative analysis of the number of neurons in cortex, CA1, CA3 and dentate gyrus (per 0.01m^2^). All data are presented as mean ± SD. n = 3.


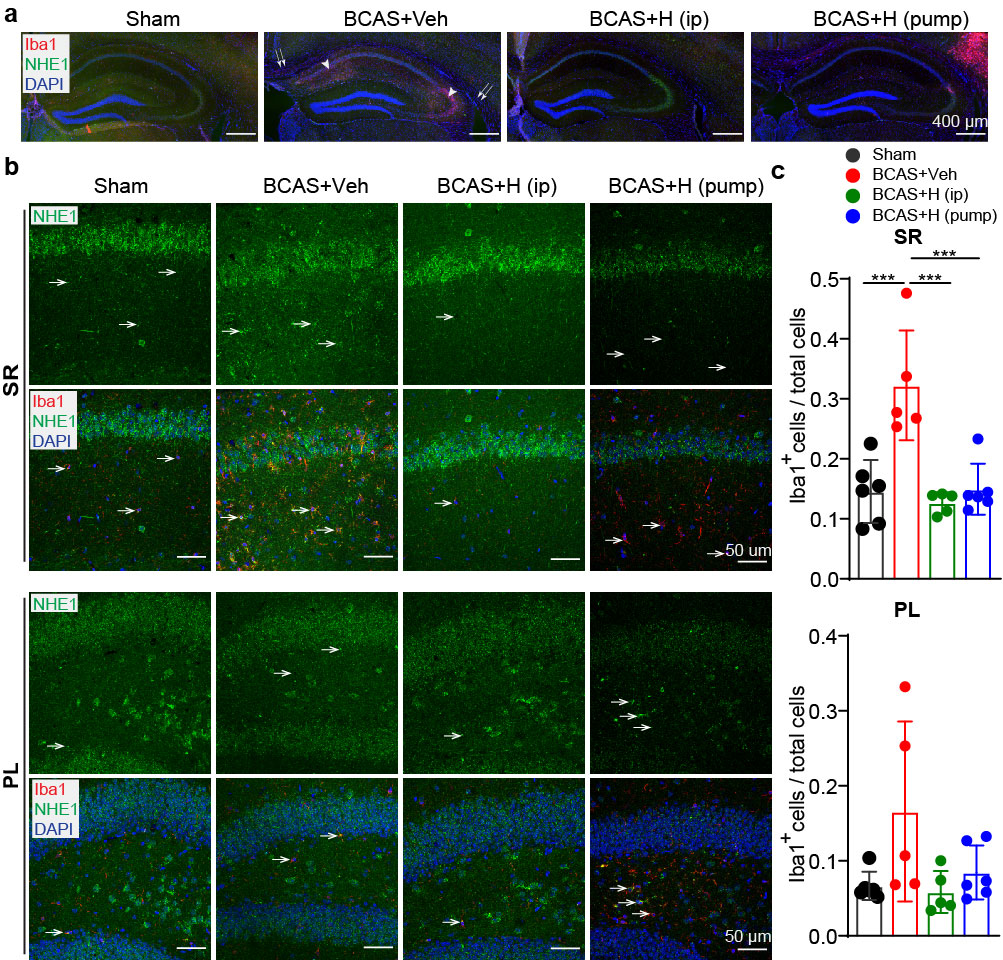


**Figure S5.** **HOE642 suppressed microglial activation in hippocampus of BCAS mice at 30d post-surgery.** (a) Representative staining images of hippocampus for NHE1 (green) and Iba1 (red) at 30d post-surgery. Double arrows: increased Iba1^+^ microglia in CC and EC. Arrow heads: increased Iba1^+^ microglia in hippocampus. Magnification X10, Scale bar=400 µm. (b) Representative immunostaining images of NHE1 (green) and Iba1 (red) in SR and PL of hippocampus at 30d post-surgery. Magnification X40, Scale bar=50 µm. White Arrows: the Iba1^+^ microglia with NHE1 expression. (c) Quantitative analysis of Iba1^+^ cells /total cells in SR and PL of hippocampus. All data are presented as mean ± SD. n=5-6. ***P < 0.001.


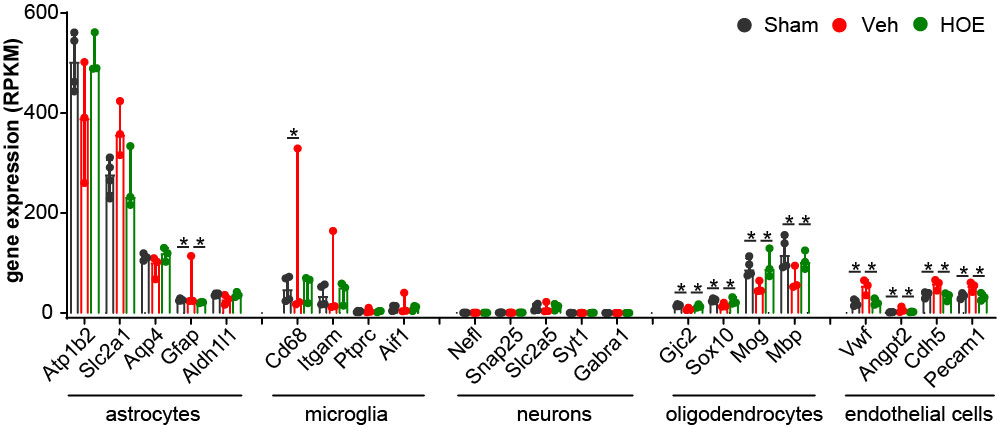


**Figure S6.** **Purity of isolated brain ACSA2^+^ astrocytes assessed by cell specific gene expression analysis.** Expression of astrocyte, microglia, neuron, oligodendrocyte and endothelial cell marker genes in the astrocytes isolated from Sham, BCAS+Veh (i.p.), and BCAS+HOE642 (i.p.) brains at 30d post-surgery. Data are RPKM values, which shown as median and interquartile range. n = 3-4, *p ≤ 0.05 and FC > 1.5

**
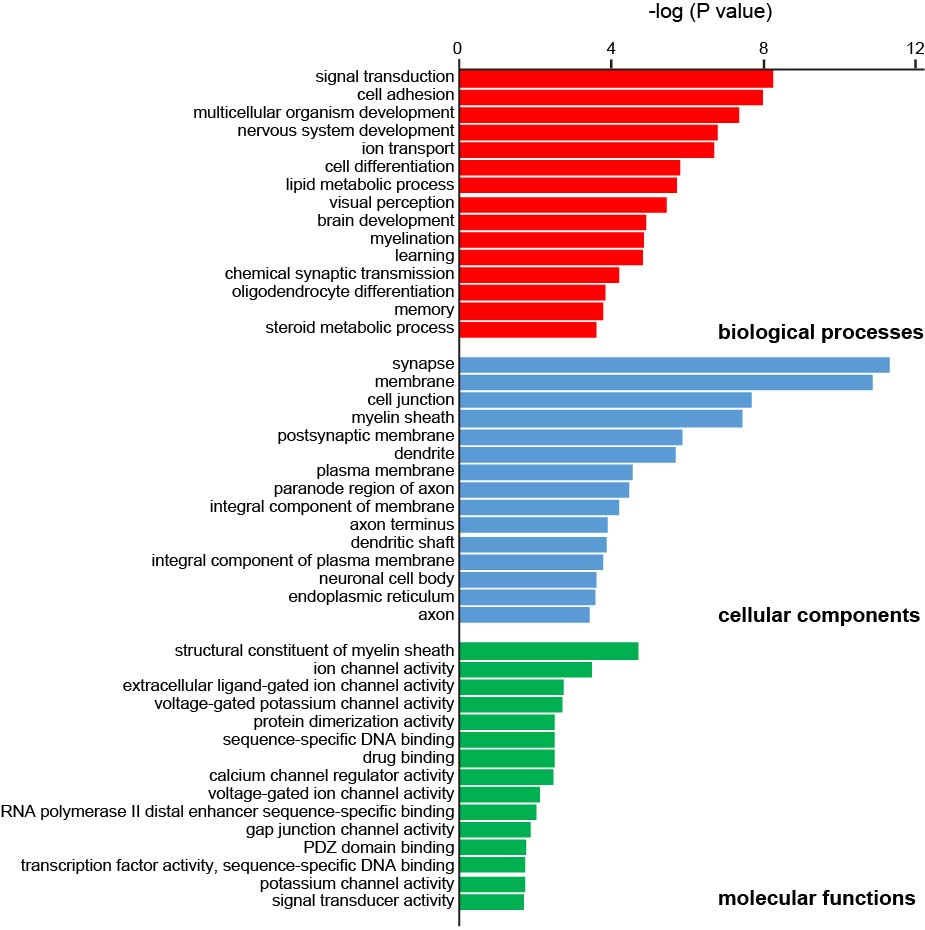
**

**Figure S7.** **The top 15 enriched GO terms of upregulated genes in astrocytes of the** **BCAS+HOE642-treated brains, compared to the BCAS+Veh brains.** The three GO categories [cellular component, biological process and molecular function] were detected using DAVID, which are statistically significant with p < 0.05 and a gene count ≥ 2 as the thresholds to indicate a difference.


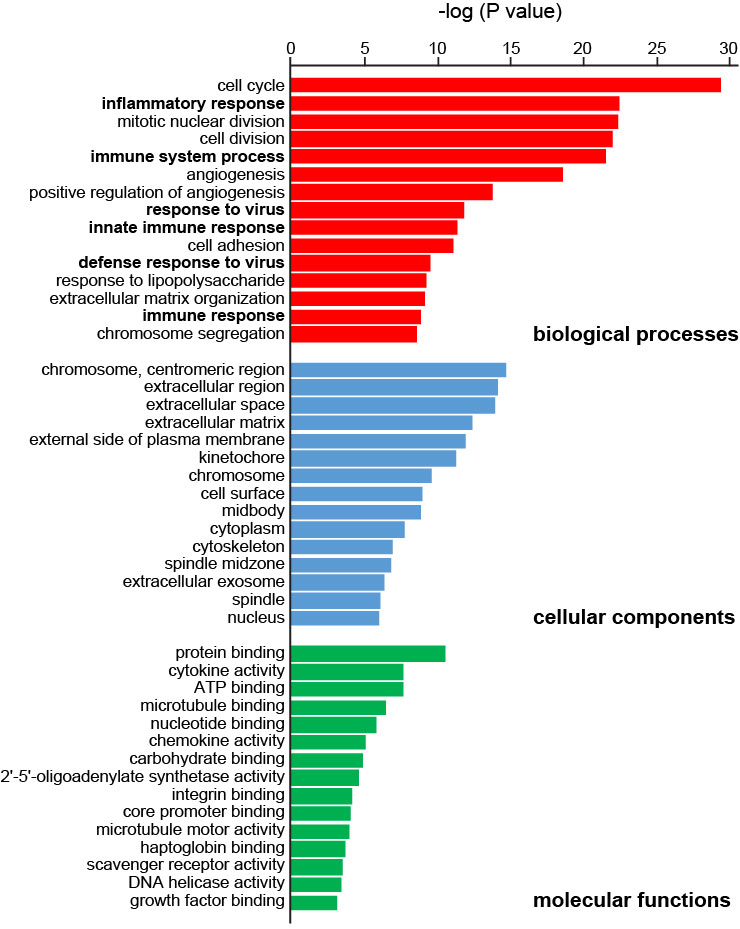


**Figure S8. The top 15 enriched GO terms of downregulated genes in astrocytes of the BCAS+HOE642-treated brains, compared to the BCAS+Veh brains.** The three GO categories [cellular component, biological process and molecular function] were detected using DAVID, which are statistically significant with p < 0.05 and a gene count ≥ 2 as the thresholds to indicate a difference.


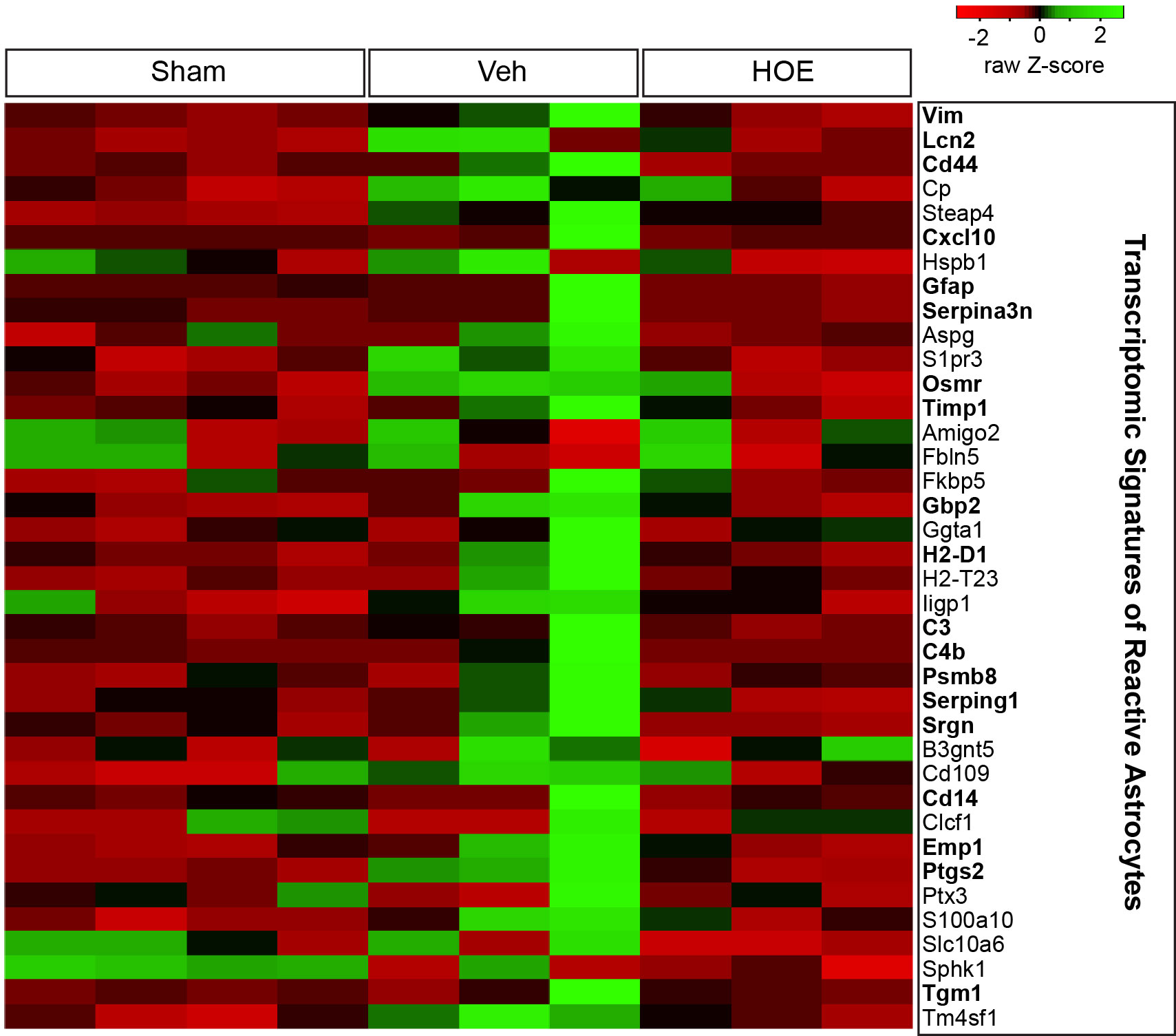


**Figure S9. Changes of astrocyte transcriptomes in Sham, BCAS+Veh, and BCAS+HOE642 groups.** Heatmap of transcriptomic signatures of reactive astrocyte was created with the HEATMAPPER [1]. Z scores of RPKM values of these genes were displayed in the heatmap. n = 3-4. Genes with DEG (p ≤ 0.05 and FC ≥ 1.5) between HOE642 vs. Veh were indicated with bold font.


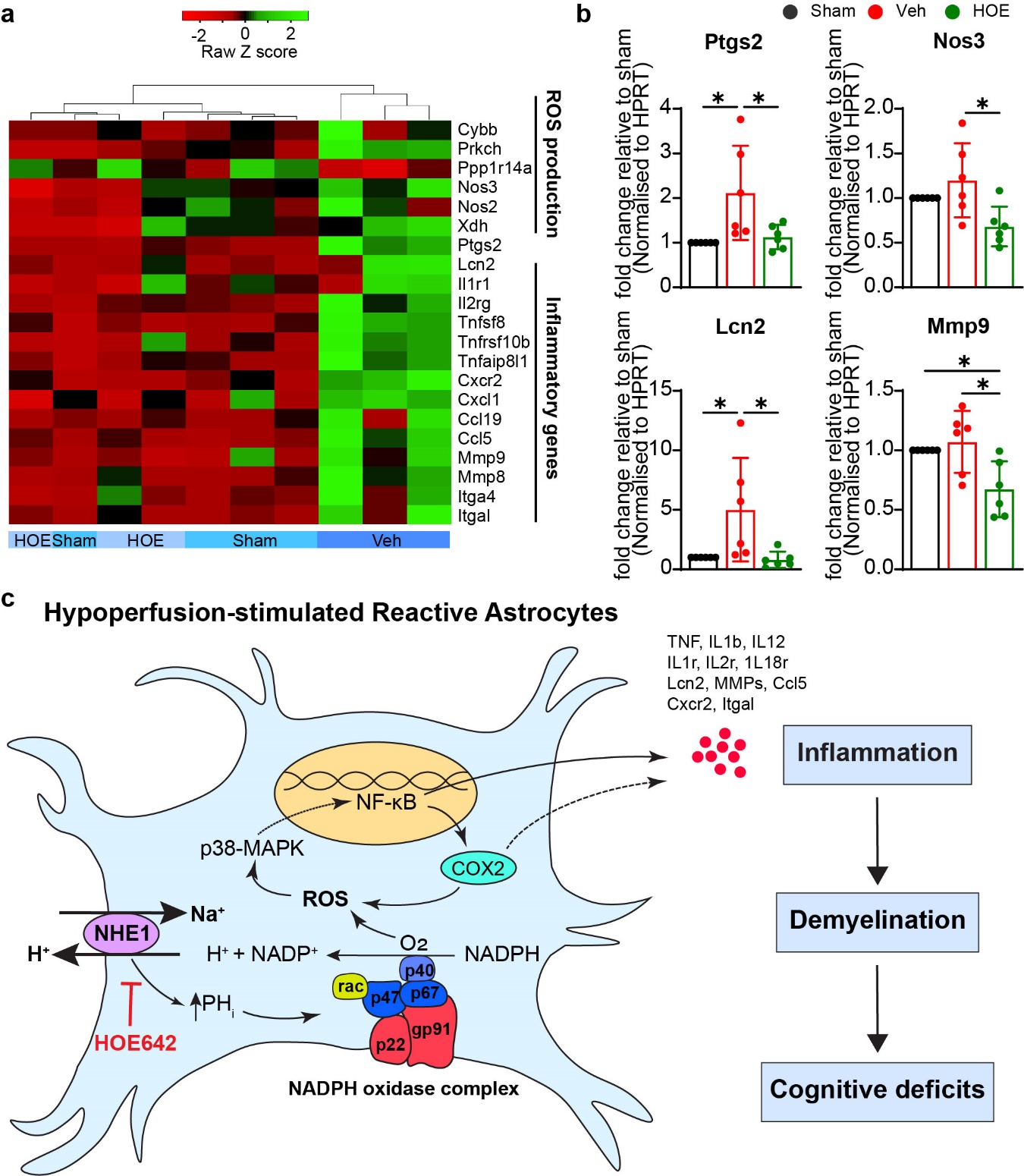


**Figure S10. HOE642 treatment suppressed upregulation of ROS production and pro-inflammatory gene transcriptomes in astrocytes of BCAS mice.** (a) Heatmap of differentially expressed ROS production genes and inflammatory genes in Sham, BCAS+Veh, and BCAS+HOE642 groups. Heatmap was created with the HEATMAPPER [1], using a complete linkage as a clustering method and the Euclidean as a distance measurement method. Z scores of RPKM values of these ROS production genes and inflammatory genes were displayed in the heatmap. n = 3-4. (b) qRT-PCR quantification of Ptgs2, Nos3, Lcn2, Mmp9. Data are mean ± SD, n = 6. *p < 0.05. (c) Schematic summary of NHE1 protein involved in ROS signaling and pro-inflammatory cytokine release in reactive astrocytes in response to cerebral hypoperfusion.

**Table S1:** Pearson correlation coefficient r between spatial working cognition, microstructural and pathologic change in white matter tracts and hippocampus.

| Corpus callosum | | | | | | | | | | | | | |
| --- | --- | --- | --- | --- | --- | --- | --- | --- | --- | --- | --- | --- | --- |
| Variables | FA | MD | AD | RD | alternation | | GFAP | IBA1 | MBP | NF | - | |  |
| alternation | .473* | -0.306 | 0.012 | -.457* | 1 | | -.733** | -.622** | .474* | 0.278 | - | |  |
| GFAP | -.558* | 0.107 | -0.28 | 0.355 | -.733** | | 1 | .554* | -0.293 | -0.398 | - | |  |
| IBA1 | -0.247 | -0.02 | -0.227 | 0.13 | -.622** | | .554* | 1 | -0.221 | -0.295 | - | |  |
| MBP | 0.227 | -0.118 | 0.04 | -0.2 | .474* | | -0.293 | -0.221 | 1 | .552* | - | |  |
| NF | .663** | -0.32 | 0.09 | -.533* | 0.278 | | -0.398 | -0.295 | .552* | 1 | - | |  |
| External capsule | | | | | | | | | | | | | |
| Variables | FA | MD | AD | RD | alternation | | GFAP | IBA1 | MBP | NF | - | |  |
| alternation | .640** | -0.284 | 0.03 | -.475* | 1 | | -.609** | -.653** | 0.446 | 0.34 | - | |  |
| GFAP | -.623** | 0.152 | -0.137 | 0.344 | -.609** | | 1 | 0.36 | -0.338 | -0.245 | - | |  |
| IBA1 | -.699** | 0.328 | -0.02 | .537* | -.653** | | 0.36 | 1 | -0.188 | -.563* | - | |  |
| MBP | 0.375 | -0.085 | 0.108 | -0.215 | 0.446 | | -0.338 | -0.188 | 1 | 0.427 | - | |  |
| NF | .805** | -0.114 | 0.277 | -0.386 | 0.34 | | -0.245 | -.563* | 0.427 | 1 | - | |  |
| Hippocampus | | | | | | | | | | | | | |
|  | FA | MD | AD | RD | alternation | | GFAP^#^ | Iba1^#^ | GFAP^§^ | Iba1^§^ | - | |  |
| alternation | 0.3 | -0.27 | -0.095 | -0.355 | 1 | | -.468* | -.835** | -.607** | -.543* | - | |  |
| GFAP^#^ | 0.123 | -0.058 | 0.011 | -0.099 | -.468* | | 1 | 0.318 | .518* | 0.336 | - | |  |
| Iba1^#^ | -.586** | 0.243 | -0.081 | 0.437 | -.835** | | 0.318 | 1 | .644** | .787** | - | |  |
| GFAP^§^ | -0.2 | 0.276 | 0.133 | 0.337 | -.607** | | .518* | .644** | 1 | .573* | - | |  |
| Iba1^§^ | -.570* | 0.157 | -0.153 | 0.354 | -.543* | | 0.336 | .787** | .573* | 1 | - | |  |
| Strata of hippocampus | | | | | | | | | | | | |  |
|  | FA^sr^ | MD^sr^ | AD^sr^ | RD^sr^ | FA^slm^ | MD^slm^ | AD^slm^ | RD^slm^ | FA^ml^ | MD^ml^ | AD^ml^ | RD^ml^ |  |
| alternation | -0.124 | 0.169 | 0.056 | 0.21 | 0.253 | -.518* | -0.41 | -.544* | 0.384 | -0.403 | 0.103 | -.464* |  |

*: Correlation is significant at the 0.05 level (2-tailed).

**: Correlation is significant at the 0.01 level (2-tailed).

^#^: In stratum radiatum of hippocampus.

^§^: In polymorph layer of dentate gyrus.

sr: stratum radiatum; slm: stratum lacunosum moleculare; ml: molecular layer of dentate gyrus (ml).

**Reference:**

1. Babicki, S., et al., *Heatmapper: web-enabled heat mapping for all.* Nucleic Acids Res, 2016. **44**(W1): p. W147-53.
